# Anti-Inflammatory Role of TRPV4 in Human Macrophages

**DOI:** 10.4049/immunohorizons.2200100

**Published:** 2023-01-16

**Authors:** Yukiko Atsumi, Manami Toriyama, Hiroko Kato, Motoki Nakamura, Akimichi Morita, Masayuki Takaishi, Kaori Saito, Miku Tanaka, Fumihiro Okada, Makoto Tominaga, Ken J. Ishii, Fumitaka Fujita

**Affiliations:** * Graduate School of Pharmaceutical Sciences, Osaka University, Osaka, Japan;; † Center for Vaccine and Adjuvant Research, National Institutes of Biomedical Innovation, Health and Nutrition, Osaka, Japan;; ‡ Graduate School of Science and Technology, Nara Institute of Science and Technology, Nara, Japan;; § Department of Geriatric and Environmental Dermatology, Graduate School of Medical Sciences, Nagoya City University, Nagoya, Japan;; ¶ Mandom Corporation, Osaka, Japan;; ‖ Exploratory Research Center on Life and Living Systems, National Institutes of Natural Sciences, Okazaki, Japan;; # National Institute for Physiological Sciences, National Institutes of Natural Sciences, Okazaki, Japan;; ** Laboratory of Vaccine Science, WPI Immunology Frontier Research Center, Osaka University, Osaka, Japan; and; †† Division of Vaccine Science, The Institute of Medical Science, The University of Tokyo, Tokyo, Japan

## Abstract

The pathology of skin immune diseases such as atopic dermatitis is closely related to the overproduction of cytokines by macrophages. Although the pathological functions of macrophages in skin are known, mechanisms of how they detect the tissue environment remain unknown. TRPV4, a nonselective cation channel with high Ca^2+^ permeability, is activated at physiological temperatures from 27 to 35°C and involved in the functional control of macrophages. However, the relationship between TRPV4 function in macrophages and skin immune disease is unclear. In this study, we demonstrate that TRPV4 activation inhibits NF-κB signaling, resulting in the suppression of IL-1β production in both human primary monocytes and macrophages derived from human primary monocytes. A TRPV4 activator also inhibited the differentiation of human primary monocytes into GM-CSF M1 macrophages but not M-CSF M2 macrophages. We also observed a significant increase in the number of inducible NO synthase–positive/TRPV4-negative dermal macrophages in atopic dermatitis compared with healthy human skin specimens. Our findings provide insight into the physiological relevance of TRPV4 to the regulation of macrophages during homeostasis maintenance and raise the potential for TRPV4 to be an anti-inflammatory target.

## Introduction

Immune cells, including neutrophils, monocytes, B cells, and T cells, contribute to homeostasis by protecting the host from bacterial and viral infections. Among these immune cells, macrophages differentiate from monocytes to engulf and digest foreign substances through phagocytosis to eliminate them (1, 2). Macrophages also produce chemokines and cytokines that attract and activate other immune cells (3). Recently, macrophages were categorized as either classically activated M1 macrophages or alternatively activated M2 macrophages, which have different cytokine expression patterns (4). Macrophages can change their phenotypes to have physiological plasticity in response to extracellular immune signals (2). M1 macrophages express inflammatory cytokines, whereas M2 macrophages produce inhibitory cytokines such as IL-10, which is involved in tissue repair (2).

M2 macrophages dominate in the healthy skin (5). Although the physiological relevance of macrophage subtypes is not completely understood, it is accepted that several subtypes of macrophages, such as Langerhans cells and dermal macrophages, are present in the skin (6). In healthy skin, macrophages help maintain homeostasis; however, they are also involved in the development of inflammatory diseases, such as contact (7) and allergic dermatitis (8). In typical allergic dermatitis, or atopic dermatitis (AD), macrophages accumulate in lesions (9). The Th2 cytokine GM-CSF, which initiates M1 macrophage differentiation, is overproduced in AD (10). Other macrophage-derived cytokines, including IL-1β (11) and IL-10 (12), are also overproduced in AD lesions, which leads to the pathology of AD and a continued Th2 response. Macrophages thus have pathological functions during AD development and progression, but the mechanisms by which the extracellular environment and/or cellular signals regulate macrophage function are not fully understood.

Transient receptor potential (TRP) channels are nonselective, cation channels that can respond to chemical and physical stimuli such as temperature, mechanical stimuli, and potential change. In 1997, the capsaicin receptor TRPV1 was discovered; TRPV1 directly senses heat and activates (13), attracting great attention as a receptor involved in sensory perception. As of 2022, 27 TRP channels were identified and several receptor functions in humans reported. In addition to inherited diseases from TRP gene mutations, spontaneous TRP channel abnormalities can lead to the development of acquired diseases and cancers (14–17), which suggests the importance of TRP channels for maintaining homeostasis in several tissues. TRPV4 has a high Ca^2+^ permeability that is activated at 27 to 35°C (18) and ubiquitously expressed throughout the body, for instance, by skin, lung, brain, and immune cells. As shown in several studies, TRPV4 helps maintain homeostasis in these tissues, such as the skin barrier (19, 20), articular cartilage (21), and lung (22). Another report indicates a function for TRPV4 in immune regulation. Macrophages derived from a TRPV4-knockout mouse increased their production of the inflammatory cytokine IL-1β while decreasing the anti-inflammatory cytokine IL-10 (23), which suggests a relationship between TRPV4 and cytokine expression. However, due to the difficulty of isolating macrophages from human tissues, the physiological function of TRPV4 in human macrophages remains unclear.

In this study, we screened the expression levels of TRP channels in immune cells using a protein atlas database and found that TRPV4 is highly expressed in monocytes, the macrophage precursors. Therefore, we evaluated TRPV4 function in both macrophages and monocytes by investigating cytokine expression and macrophage differentiation.

## Materials and Methods

### Isolating human monocytes

All studies involving human subjects were conducted in accordance with the guidelines of the World Medical Association’s Declaration of Helsinki. The ethical review for medical and health research involving human subjects was conducted and approved by Osaka University (approval number 2019-1) and Nagoya City University (approval number 60-18-0003). All experiments followed the applicable ethical guidelines of these facilities. Whole peripheral blood samples for PBMCs were purchased from the Japanese Red Cross Society (JRCS) according to the *Guidelines on the Use of Donated Blood in Research and Development*, etc. We purchased 164 samples from 16- to 69-y-old healthy volunteers whose donations were too small for use as blood products by the JRCS. PBMCs were isolated from the whole peripheral blood using Ficoll-Paque PLUS (GE Healthcare) and density-gradient centrifugation. To lyse erythrocytes, they were incubated with ACK buffer (150 mM NH_4_Cl, 10 mM KHCO_3_, and 0.1 mM EDTA) for 15 min at room temperature. Dead cells were removed using a Dead Cell Removal Kit (130-042-401; Miltenyi Biotec). The isolated PBMCs were labeled with CD14 microbeads (130-050-201; Miltenyi Biotec), and a CD14-positive cell population was isolated using magnetic separation, which we considered to be monocytes.

### Cell culture

Monocytes were cultured in RPMI 1640 medium (11875-093; Life Technologies) containing 20% FBS (172012; Sigma-Aldrich). HEK293 cells stably expressing human TLR4a, MD2, and CD14 genes (293/hTLR4-MD2-CD14; InvivoGen) were cultured in DMEM high-glucose (D6429; Sigma-Aldrich) medium containing 10% FBS, 100 µg/ml normocin (ant-nr; InvivoGen), 10 µg/ml blasticidin (anti-bl; InvivoGen), and 50 µg/ml Hygromycin B Gold (ant-hg; InvivoGen). Cells were passaged every 2 to 3 d, when they reached 80% confluency.

### Macrophage differentiation

Monocytes were cultured for 7 d in RPMI 1640 medium containing 20% FBS and 1% antibiotics (antibiotic-antimycotic; Life Technologies). To differentiate monocytes into M1 macrophages, they were supplemented with 50 ng/ml GM-CSF (100-08; Shenandoah Biotechnology) for 7 d. Monocytes were supplemented with 50 ng/ml M-CSF (100-03; Shenandoah Biotechnology) for differentiation into M2 macrophages. Fresh medium was added on days 2 and 5 of the incubation. GM-CSF and M-CSF macrophages, as appropriate, were defined as adherent cells after 7 d of culturing (24).

### Inflammatory skin disease samples

Formalin-fixed, paraffin-embedded skin sections were obtained from patients following the ethical guidelines of Nagoya City University (approval number 60-18-0003).

### Quantitative RT-PCR

To confirm the expression of TRPV4 mRNA, total mRNA was isolated from monocytes (day 0), monocytes cultured for 7 d (day 7 monocytes), GM-CSF macrophages, and M-CSF macrophages. Cells were washed with PBS, and the total RNA was extracted with TRI Reagent (TR 118; Molecular Research). The extracted RNA was stored at −80°C until analysis.

To confirm the mRNA expression of IL-1β, 5.0 × 10^5^ cells/ml monocytes or 10^5^ cells/ml macrophages were cultured for 6 h in RPMI 1640 medium containing 0.5% FBS. The cells were pretreated for 30 min with 30 µM of the TRPV4 inhibitor HC067047 (4100; Tocris Bioscience) and then stimulated with LPS (L2630; Sigma-Aldrich) to induce IL-1β. Monocytes were costimulated with 100 µM of GSK1016790A (073-06491; FUJIFILM Wako Chemicals), a TRPV4 activator, and 10 pg/ml LPS. GM-CSF or M-CSF macrophages were stimulated with 10 µM GSK1016790A and 10 ng/ml LPS. Six hours after stimulation, cells were washed with PBS, and the total RNA was extracted.

The RNA sample was reverse transcribed to cDNA using the QuantiTect Reverse Transcription Kit (205315; Qiagen) and the cDNA sequence amplified using THUNDERBIRD SYBR qPCR Mix (QPS-201; TOYOBO). The primers used are as follows: TRPV4, forward 5′-CTACGCTTCAGCCCTGGTCTC-3′ and reverse 5′-GCAGTTGGTCTGGTCCTCATTG-3′; 18s RNA, forward 5′- CGGCTACCACATCCAAGGAA-3′ and reverse 5′-AGCTGGAATTACCGCGGC-3′; IL-1β, forward 5′-CACGATGCACCTGTACGATCA-3′ and reverse 5′-GTTGCTCCATATCCTGTCCCT-3′; and GAPDH, forward 5′-CATCCCTGCCTCTACTGGCGCTGCC-3′ and reverse 5′-CCAGGATGCCCTTGAGGGGGCCCTC-3′.

### Western blotting

As a positive control for TRPV4 expression, HEK293T cells were transfected with a pcDNA3.1 vector encoding human TRPV4 using polyethylenimine (24765-100; Polysciences). Prior to performing the Western blots, 10^6^ monocytes were differentiated into either GM-CSF or M-CSF macrophages and then treated with reagents. For NLRP3 and caspase-1 expression, the cells were pretreated with 30 µM HC067047 for 30 min and then stimulated with 10 ng/ml LPS and 10 µM GSK1016790A for 6 h in 1 ml of RPMI 1640 medium supplemented with 0.5% FBS. For phosphorylated and total IκBα and NF-κB, cells were stimulated with 10 ng/ml LPS and 10 µM GSK1016790A for 15 min, 30 min, 1 h, 6 h, and 24 h in 1 ml of RPMI medium containing 0.5% FBS.

Cells were prepared for lysis with a PBS wash followed by RIPA Lysis Buffer (sc-24948; Santa Cruz Biotechnology) containing protease inhibitor (cOmplete mini; Roche) and phosphatase inhibitor (PhosSTOP; Roche). Lysates were mixed with Laemmli Sample Buffer (Bio-Rad Laboratories), sonicated, and boiled at 95°C for 10 min prior to gel loading. Proteins were detected using the following Abs: anti-TRPV4 (1:100) and anti-NLRP3 (1:500) from Abcam (ab39260 and ab263899, respectively); anti–caspase-1 (1:1,000), anti-IκBα (1:500), anti-phosphorylated NF-κB (1:1,000), and anti–NF-κB (1:1,000) from Cell Signaling Technology (#3866, #9246S, #3036, and #6956, respectively); and anti-phosphorylated IκBα (c-21; 1:1,000) as well as anti–β-actin (1:1000) from Santa Cruz Biotechnology (sc371 and sc-1616, respectively).

### Multiplex cytokine detection

To detect cytokines, we ran a ProcartaPlex 20-cytokine human inflammation panel per the manufacturer’s instructions (EPX200-12185-901; Thermo Fisher Scientific). To stimulate cytokine production, 5.0 × 10^5^ cells/ml monocytes were stimulated with 10 pg/ml LPS and 100 µM GSK1016790A in RPMI medium supplemented with 0.5% FBS. After a 6-h incubation, 0.1% (v/v) Triton X-100 was added to lyse the cells. The cell lysate was centrifuged and the supernatant used for cytokine detection.

### ELISA cytokine detection

In preparation for ELISA-based cytokine detection, 5.0 × 10^5^ cells/ml monocytes were cultured and stimulated in RPMI 1640 medium supplemented with 0.5% FBS. After a 30-min pretreatment of 30 µM HC067047, cells were stimulated with 10 pg/ml LPS for 6 h. IL-1β was detected in 1, 10, and 100 µM GSK1016790A-treated cells, whereas IL-1α, IL-6, and TNF-α were measured following a 100-µM GSK1016790A stimulation.

GM-CSF and/or M-CSF macrophages were collected and treated with TrypLE Express (12604-013; Life Technologies) at 37°C for 15 min. Macrophages were cultured at 10^5^ cells/ml in a 24-well plate with 400 µl of RPMI 1640 medium supplemented with 0.5% FBS. After a 30-min pretreatment with 30 µM HC067047, cells were costimulated with 10 ng/ml LPS and 10 µM GSK1016790A for 6 h to detect IL-1β. For IL-10 and IL-8 detection, cells were stimulated for 24 h. To confirm the effect of FK506 (10007965; Cayman Chemical) by measuring IL-1β, macrophages were pretreated with 1 nM FK506 for 30 min. After pretreatment, cells were cotreated with 10 ng/ml LPS and 10 µM GSK1016790A for 6 h.

Cell lysate was prepared by adding 0.1% (v/v) Triton X-100 to the cell culture directly and then stored at 4°C until analysis. The lysate was centrifuged and the supernatant used for the assay. R&D Systems Human Duo Set ELISA kits were used to detect the following cytokines: IL-1β, IL-1α, IL-6, TNF-α, IL-10, and IL-8 (DY201-05, DY200-05, DY206-05, DY210-05, DY217B-05, and DY208-05, respectively). The absorbance (excitation 450 nm) was measured using a microplate reader (Infinite F200 PRO; Tecan); the background absorbance was measured at 560 nm.

### Ca^2+^ imaging

Cytosolic Ca^2+^ imaging was performed as previously described (25, 26). GM-CSF or M-CSF macrophages on coverslips were mounted in an open chamber and perfused with standard bath solution (140 mM NaCl, 5 mM KCl, 2 mM MgCl_2_, 2 mM CaCl_2_, 10 mM HEPES, and 10 mM glucose [pH 7.4]). Cytosolic free Ca^2+^ concentrations in macrophages were measured by dual-wavelength fura 2 (Molecular Probes, Invitrogen) microfluorometry with excitation at 340/380 nm and emission at 510 nm. The fura 2 ratio image was calculated and acquired using the IP-Lab imaging processing system (Scanalytics Inc, Fairfax, VA).

### RNA interference

Human small interfering RNA (siRNA) targeting human TRPV4 (s34001, 4392421) and a negative control (#2, 439087) were purchased from Ambion. GM-CSF macrophages were transfected with 75 nM siRNA by electroporation (Neon Transfection system; Invitrogen). After electroporation, cells were incubated for 2 d in RPMI supplemented with 20% FBS and 10^5^ cells/ml cultured in a 24-well plate with 400 µl of RPMI 1640 medium supplemented with 0.5% FBS. The cells were then costimulated with 10 ng/ml LPS and 10 µM GSK1016790A for 6 h to detect IL-1β using ELISA. M-CSF macrophages were cultured at 5.0 × 10^4^ cells/well in a 24-well plate and then transfected with 100 nM siRNA by Lipofectamine RNAiMAX (13778-150; Invitrogen) 24 h after passage per the manufacturer’s instructions. Six hours after the transfection, the media was replaced with RPMI 1640 medium supplemented with 20% FBS. Two days after transfection, the cells were suspended in 400 µl of RPMI 1640 medium plus 0.5% FBS and either costimulated with 10 ng/ml LPS and 10 µM GSK1016790A for 6 h to detect IL-1β or stimulated for 24 h to detect IL-10. The knockdown efficiency in both macrophages was confirmed by quantitative RT-PCR (RT-qPCR).

### Luciferase assay

293/hTLR4-MD2-CD14 cells were cultured in a 24-well plate and cotransfected with NanoLuc experimental reporter (pNL3.2.NF-κB-RE[NlucP/NF-κB-RE/Hygro] vector or pNL1.2[NlucP] vector; Promega) and firefly loading control reporter (pGL4.54[luc2/TK] vector; Promega) with pcDNA3.1-TRPV4 or the pcDNA3.1 empty vector with polyethylenimine. Promoter response elements (REs) were inserted into pNL1.2[NlucP] at the KpnI/HindIII site. Cells were cultured for 24 h in DMEM medium plus 10% FBS after transfection. Twenty-four hours after transfection, the culture medium was replaced with 500 µl of DMEM medium plus 0.5% FBS, 1 µg/ml LPS, and 50 µM GSK1016790A. Twenty-four hours later, luminescence was detected using a Nano-Glo Dual-Luciferase Reporter Assay System (Promega) per the manufacturer’s instructions.

To confirm the effect of FK506, 293/hTLR4-MD2-CD14 cells were transfected with the NanoLuc, NFAT-RE, and firefly reporters as well as the pcDNA3.1-TRPV4 vector. Cells were stimulated for 24 h with 50 µM GSK1016790A and 1 nM FK506. Promoter REs inserted into the pNL1.2[NlucP] vector were amplified using the following primers: NFAT, forward 5′-GGAGGAAAAACTGTTTCATACAGAAGGCGTGGAGGAAAAACTGTTTCATACAGAAGGCGTGGAGGAAAAACTGTTTCATACAGAAGGCGT-3′ and reverse 5′-ACGCCTTCTGTATGAAACAGTTTTTCCTCCACGCCTTCTGTATGAAACAGTTTTTCCTCCACGCCTTCTGTATGAAACAGTTTTTCCTCC-3′; PU.1, forward 5′-TACTCTTTTCCCCTTTCCTTTAACT-3′ and reverse 5′-AGTTAAAGGAAAGGGGAAAAGAGTA-3′; AP-1, forward 5′-TGAGTCAGTGACTCAGTGAGTCAGTGACTCAGTGAGTCAGTGACTCAG-3′ and reverse 5′-CTGAGTCACTGACTCACTGAGTCACTGACTCACTGAGTCACTGACTCA-3′; CREB, forward 5′-GCACCAGACAGTGACGTCAGCTGCCAGATCCCATGGCCGTCATACTGTGACGTCTTTCAGACACCCCATTGACGTCAATGGGAGAAC-3′ and reverse 5′-GTTCTCCCATTGACGTCAATGGGGTGTCTGAAAGACGTCACAGTATGACGGCCATGGGATCTGGCAGCTGACGTCACTGTCTGGTGC-3′; and IRF5, forward 5′-GCCTAGCACTAACCGAAACCGAAACCTAAGTGCTA-3′ and reverse 5′-TAGCACTTAGGTTTCGGTTTCGGTTAGTGCTAGGC-3′.

### Flow cytometry

To investigate the effect of TRPV4 activation on the differentiation of monocytes into macrophages, 10 µM GSK1016790A was added to 50 ng/ml GM-CSF or 50 ng/ml M-CSF and cultured for 7 d. GSK2193874 (SML0942-5MG; Sigma-Aldrich), an antagonist of TRPV4, was added at 1 µM simultaneously. After 7 d, the adherent cells were collected by incubating them with TrypLE Express at 37°C for 15 min. Dead cells were stained with LIVE/DEAD Fixable Aqua Dead Cell Stain kit (L34966; Invitrogen) and the cell-surface marker CD11b stained with allophycocyanin-conjugated anti-human CD11b Ab (301309; BioLegend). After labeling, the cells were fixed with 0.5% (v/v) paraformaldehyde, and protein expression was analyzed using a BD FACSAria II (BD Biosciences). All data were analyzed by using BD FACSDiva and FlowJo software.

### Fluorescence in situ hybridization and immunostaining

All formalin-fixed, paraffin-embedded skin sections were obtained from patients or healthy volunteers following the ethical guidelines of Nagoya City University (approval number 60-18-0003). Skin samples were deparaffinized and pretreated using the RNAscope Multiplex Fluorescent Reagent Kit v2 (323100; ACDBio) per the manufacturer’s protocol. Fluorescence in situ hybridization (FISH) was performed using a human TRPV4 probe (RNAscope Target Probe Hs-TRPV4, 452221; ACDBio), detected by Opal 520 (Opal 520 Reagent Pack, FP1487001KT; Akoya Biosciences) (1:1,500), and followed by DAPI staining. Positive (RNAscope Positive Control Probe Hs-PPIB, 313901; ACDBio) and negative probes (RNAscope Negative Control Probe-DapB, 310043; ACDBio) were used as controls in each experiment. Slides were blocked by incubation in 10% FBS and 0.1% Triton X-100 in PBS for 30 min at room temperature. Primary Abs were incubated overnight at 4°C. The following Abs were used: anti-CD11b (1:100; ab52478; Abcam); Alexa Fluor 647–conjugated anti-arginase-1 (1:100; #43279; Cell Signaling Technology); allophycocyanin-conjugated, anti-human CD11b (301309; BioLegend); Alexa Fluor 594–conjugated anti–inducible NO synthase (iNOS; 1:100; sc-7271; Santa Cruz Biotechnology); anti-CD68 (1:100) (#76437; Cell Signaling Technology); and anti–arginase-1 (1:100; #43933; Cell Signaling Technology). Goat anti-rabbit IgG (H+L) cross-adsorbed secondary Ab and Alexa Fluor 594 (A-11037; Thermo Fisher Scientific) were used for CD11b visualization. Goat anti-mouse IgG (H+L) cross-adsorbed secondary Ab and Alexa Fluor 594 (A-11032; Thermo Fisher Scientific) were used for arginase-1 detection. Donkey anti-rabbit IgG (H+L) cross-adsorbed secondary Ab and Alexa Fluor 647 (A-31573; Thermo Fisher Scientific) were used for CD68 detection.

Samples were mounted using a Prolong Gold (Thermo Fisher Scientific), and slides were observed using an Olympus VS200 research slide scanner. Ten magnified dermis images were taken by VS200 and the number of macrophages in each counted. To measure the intensity of expression of each marker, we set the threshold of intensity by quantifying positive and negative signals. Cells above the threshold were defined as positive, and cells below the threshold were defined as negative.

Donor information was as follows: patients with AD were a 19-y-old female, 30-y-old male, 44-y-old female, 40-y-old female, and 29-y-old male. Healthy donors were a 50-y-old male, 38-y-old female, 53-y-old female, 36-y-old male, and 40-y-old female.

### Statistical analysis

Statistical analyses were performed with Excel (Microsoft) software or GraphPad Prism 7 software (MDF). The cytokine production, mRNA expression level, and protein expression levels of NLRP3 and caspase-1 were analyzed using the Steel test. The knockdown efficiency and Ca^2+^ imaging were analyzed using the Mann–Whitney *U* test. The Wilcoxon signed-rank one-sided test was used to compare the expression level of NF-κB components induced by LPS with or without GSK1016790A. The Tukey-Kramer test was used to analyze luciferase assay results. The Kruskal-Wallis test was used to analyze the characteristics of macrophages in the skin.

## Results

### TRPV4 activation in macrophages decreases LPS-induced cytokine expression

**FIGURE 1.**
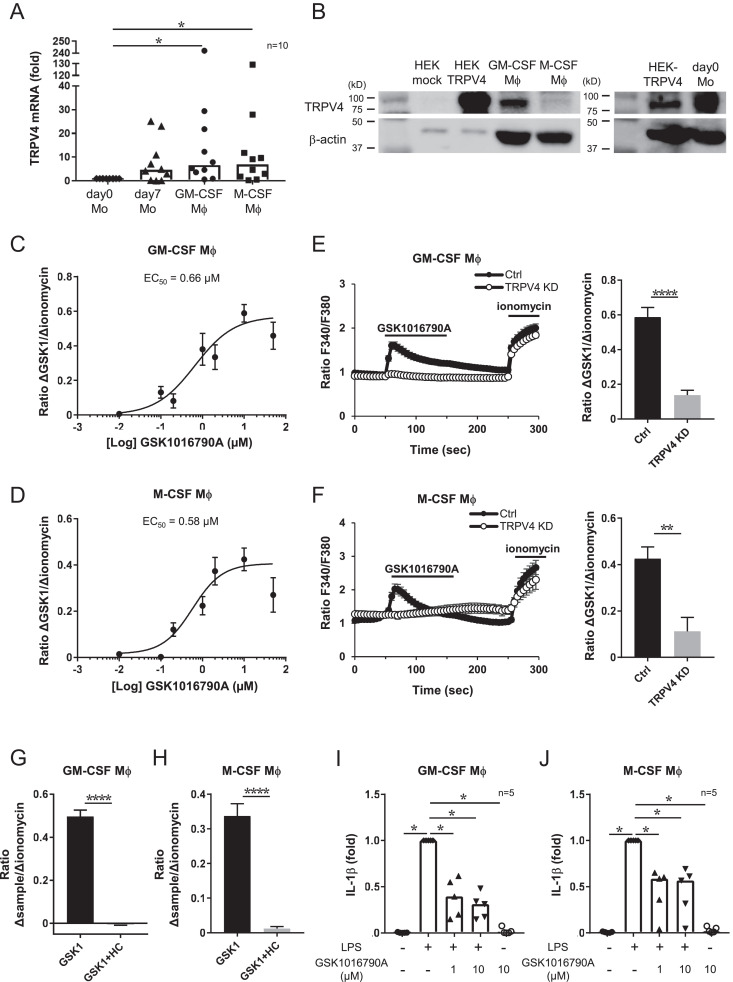
Functional expression of TRPV4 in human macrophages. (**A**) Median rate of TRPV4 mRNA expression in day 0 monocytes (Mo), day 7 monocytes, monocyte-derived GM-CSF macrophages (Mφ),m and M-CSF macrophages. Values were normalized to 18s rRNA expression. *p < 0.05, Steel test; *n* = 10 donors. (**B**) Western blot of TRPV4 (90 kDa) and β-actin (40 kDa) in GM-CSF macrophages, M-CSF macrophages, and day 0 monocytes. HEK293T cell lysate transfected with pcDNA3.1-TRPV4 as a positive control (HEK-TRPV4) or pcDNA3.1-empty as negative control (HEK-mock) was used. Dose dependence of GSK1016790A (0.01, 0.1, 0.2, 1, 2, 10, and 50 μM) assessed by measuring cytosolic Ca^2+^ increases in GM-CSF (**C**) or M-CSF (**D**) macrophages, respectively. Changes in fura 2 ratio by GSK1016790A were normalized to changes in the fura 2 ratio by 10 μM ionomycin. Data are presented as the mean ± SEM. EC_50_ values were 0.66 μM in GM-CSF macrophages (*n* = 10–49, 2 donors) and 0.58 μM in M-CSF macrophages (*n* = 6–90, 3 donors). Fura 2 ratio changes by 10 μM GSK1016790A in GM-CSF (**E**) or M-CSF (**F**) macrophages control (black) and TRPV4 knockdown cells (white) (left). (E and F) Changes in fura 2 ratio by GSK1016790A were normalized to changes in the fura 2 ratio by 10 μM ionomycin (right). Data are presented as the mean ± SEM (GM-CSF Mφ, Ctrl, *n* = 52; TRPV4 KD, *n* = 110, 2 donors; and M-CSF Mφ, Ctrl, *n* = 29, TRPV4 KD, *n* = 6, 2 donors). **p < 0.01, ****p < 0.0001, Mann–Whitney *U* test. Effects of 30 μM HC067047 on 10 μM GSK1016790A-induced cytosolic Ca^2+^ increases in GM-CSF (**G**) or M-CSF (**H**) macrophages. Changes in fura 2 ratios by GSK1016790A or GSK1016790A plus HC067047 were normalized to changes of the fura 2 ratio by 10 μM ionomycin. The bar graph shows the mean ± SEM (GM-CSF Mφ, *n* = 57, 1 donor; and M-CSF Mφ, *n* = 14, 1 donor). ****p < 0.0001, Mann–Whitney *U* test. Expression levels of IL-1β in GM-CSF (**I**) or M-CSF (**J**) macrophages were measured by ELISA. Cells were costimulated for 6 h with 10 ng/ml LPS and 1 or 10 µM GSK1016790A. All values were normalized to the LPS-alone group. Graph shows median. *p < 0.05, Steel test; *n* = 5 donors.

To investigate the mRNA expression patterns of 27 TRP channels expressed in human immune cells (including granulocytes, monocytes, dendritic cells, NK cells, B cells, and T cells), we searched and analyzed transcriptome data available from the Human Protein Atlas database (www.proteinatlas.org/). A cell cluster analysis using single-cell RNA sequence data revealed that TRPs V1, V2, V4, M2, M3, M4, M6, M7, M8, and C6 were expressed in immune cells. In monocyte clusters, the TRPs V2, V4, M2, M4, and M7 were expressed. Although most of these TRP channels were expressed in several cell populations, only TRPV4 was expressed exclusively in monocytes (ENSG00000111199-TRPV4/immune+cell). To confirm the expression of TRPV4 in human primary monocytes, we measured TRPV4 mRNA levels in human primary monocytes and monocyte-derived macrophages using RT-qPCR. Due to technical difficulties in isolating macrophages from human tissues, we isolated monocytes from PBMCs and differentiated macrophages in vitro with a 7-d GM-CSF or M-CSF cytokine stimulation. Compared to primary, day 0 monocytes, we found that TRPV4 mRNA was expressed 6.6 times higher in GM-CSF (M1) macrophages and 6.9 times higher in M-CSF (M2) macrophages (Fig. 1A). To investigate TRPV4 protein expression, we performed Western blotting analysis. As expected, a TRPV4 band was detected in all three cell types, namely day 0 monocytes, GM-CSF macrophages, and M-CSF macrophages, although we did not find significant differences between these samples (Fig. 1B, Supplemental Fig. 1A, 1B). These results suggested that human monocyte-derived macrophages and primary monocytes express TRPV4 protein.

Macrophages and monocytes secrete inflammatory cytokines when they are activated by stimuli during immune responses (4). To ask if TRPV4 activation regulates cytokine expression, we used the Bio-Plex Multiplex ELISA system and performed the first screen using primary monocytes. We stimulated monocytes with LPS for 6 h and then measured cytokine levels in the supernatant lysate mixture. This analysis identified a decrease of 17 cytokine levels in human monocytes stimulated with GSK1016790A, a specific TRPV4 activator (Supplemental Fig. 1C), which suggests that TRPV4 activation in monocytes decreases inflammatory cytokine expression. To more quantitatively measure the expression level of cytokines, we also employed multiple single ELISA analyses. Like the Bio-Plex data, IL-1β production was decreased in an inhibitor concentration-dependent manner. This decrease in expression was minimized by pretreatment with HC067047, a TRPV4 inhibitor (Supplemental Fig. 1D). Contrary to our expectations, HC067047 moderated only the effect of GSK1016790A on IL-1β expression; cotreating HC067047 and GSK1016790A did not change IL-1α, IL-6, or TNF-α levels compared with the GSK1016790A alone group, although these cytokine levels were significantly suppressed by GSK1016790A (Supplemental Fig. 1E–G). These results suggested that IL-1β regulation was TRPV4 activation–specific, so we next asked what the effect of TRPV4 activation was on IL-1β expression in monocyte-derived macrophages.

Next, we investigated whether human macrophages express TRPV4 functionally by observing cytosolic Ca^2+^. The dose dependence of GSK1016790A revealed an EC_50_ of 0.66 µM and 0.58 µM in GM-CSF and M-CSF macrophages, respectively (Fig. 1C, 1D). As we found that an increase in intracellular Ca^2+^ concentrations by 10 µM GSK1016790A was significantly suppressed in TRPV4 knockdown macrophages, we suggest that increase in intracellular Ca^2+^ concentrations induced by GSK1016790A is TRPV4-dependent (Fig. 1E, 1F). Similarly, we investigated the effect of the TRPV4 inhibitor HC067047 on an increase in intracellular Ca^2+^ concentrations, and we found that 30 µM HC067047 significantly inhibited increase in intracellular Ca^2+^ concentrations by GSK1016790A in both GM-CSF and M-CSF macrophages (Fig. 1G, 1H) (23). These data suggested that human macrophages express functional TRPV4, so we next investigated the physiological meaning of TRPV4 expression in these cells.

We examined the effect of GSK1016790A on LPS-induced IL-1β production in GM-CSF and M-CSF macrophages. Cells were costimulated with 10 ng/ml LPS and 1 or 10 µM GSK1016790A for 6 h, and IL-1β production was detected by ELISA (Fig. 1I, 1J). The results suggested that GSK1016790A significantly suppressed IL-1β production compared with stimulation with LPS alone (Fig. 1I, 1J). Based on the results of Ca^2+^ imaging and ELISA, we employed 10 µM GSK1016790A for further experiments.

**FIGURE 2.**
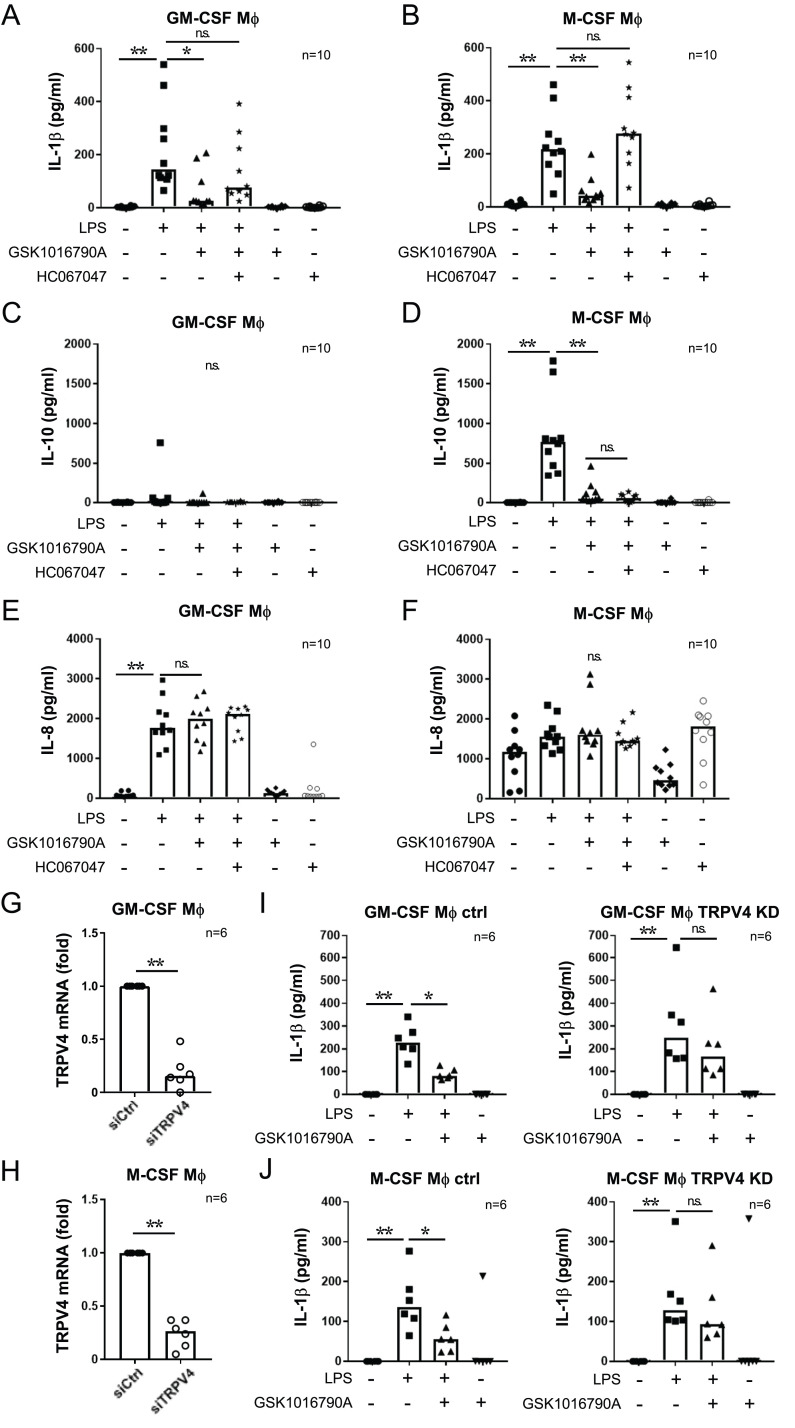
Activation of TRPV4 suppresses the production of IL-1β in macrophages. Expression of IL-1β (**A** and **B**), IL-10 (**C** and **D**), and IL-8 (**E** and **F**) in GM-CSF or M-CSF macrophages (Mφ) as measured by ELISA. Cells were costimulated for 6 (A and B) or 24 h (C–F) with 10 ng/ml LPS and 10 µM GSK1016790A after a 30-min pretreatment with 30 µM HC067047. The graph shows the median value. *p < 0.05, **p < 0.01, Steel test; *n* = 10 donors. Knockdown efficiency of TRPV4 measured by RT-qPCR in GM-CSF (**G**) and M-CSF (**H**) macrophages. The bar graph shows the median value. **p < 0.01, Mann–Whitney *U* test; *n* = 6 donors. (**I** and **J**) IL-1β expression in macrophages treated with control siRNA (left) and TRPV4 siRNA (right) measured by ELISA. Cells were costimulated with 10 ng/ml LPS and 10 µM GSK1016790A. GM-CSF (I) and M-CSF (J) macrophages were stimulated for 6 h. The bar graph shows the median values. *p < 0.05, **p < 0.01, Steel test; *n* = 6 donors.

We next stimulated macrophages with LPS for 6 h to induce cytokine expression with either a TRPV4 activator or inhibitor. In both GM-CSF and M-CSF macrophages, TRPV4 activation decreased IL-1β expression (Figs. 1I, 1J, 2A, 2B). Similar to monocytes, the effect of GSK1016790A was moderated by HC067047, suggesting that TRPV4 activation decreases IL-1β expression in both macrophages and monocytes. HC067047 alone with LPS did not change IL-1β expression (Supplemental Fig. 1H, 1I). To further understand the effect of TRPV4 on anti-inflammatory cytokine expression, we investigated IL-10, a cytokine mainly secreted by M2 macrophages. Although we did not detect IL-10 in GM-CSF macrophages (Fig. 2C), M-CSF macrophages produced IL-10 following stimulation with LPS for 24 h; the activation of TRPV4 suppressed IL-10 production (Fig. 2D). However, these effects were not inhibited by the TRPV4 inhibitor HC067047. Having shown that TRPV4 activation decreased the expression of several cytokines, including chemokines and Th2 cytokines, in monocytes (Supplemental Fig. 1C), we next investigated the expression levels of the chemokine IL-8 and the Th2 cytokines IL-4 and IL-13 in macrophages stimulated with LPS. The activation of TRPV4 did not change LPS-induced IL-8 expression (Fig. 2E, 2F). Furthermore, IL-4 and IL-13 were not detected in either macrophages (unpublished data), suggesting that TRPV4 activation specifically regulated IL-1β expression induced by LPS.

We were concerned that high concentrations of TRPV4 agonists/antagonists might affect other TRP channels (27, 28), although GSK1016790A is accepted as a highly selective TRPV4 agonist. To further validate the role of TRPV4 in decreased IL-1β, we performed knockdown experiments using siRNA to target human TRPV4 (Fig. 2G, 2H). As expected, GSK1016790A decreased IL-1β expression in nontarget siRNA-treated macrophages (Fig. 2I, 2J). In contrast, the TRPV4 knockdown minimized the effect of GSK1016790A, suggesting that the decrease of IL-1β induced by GSK1016790A is TRPV4-dependent. Altogether, our data strongly suggest that TRPV4 activation decreases LPS-induced IL-1β expression in both human M1 and M2 macrophages.

### TRPV4 activation suppresses mRNA expression of IL-1β and NLRP3 in macrophages

**FIGURE 3.**
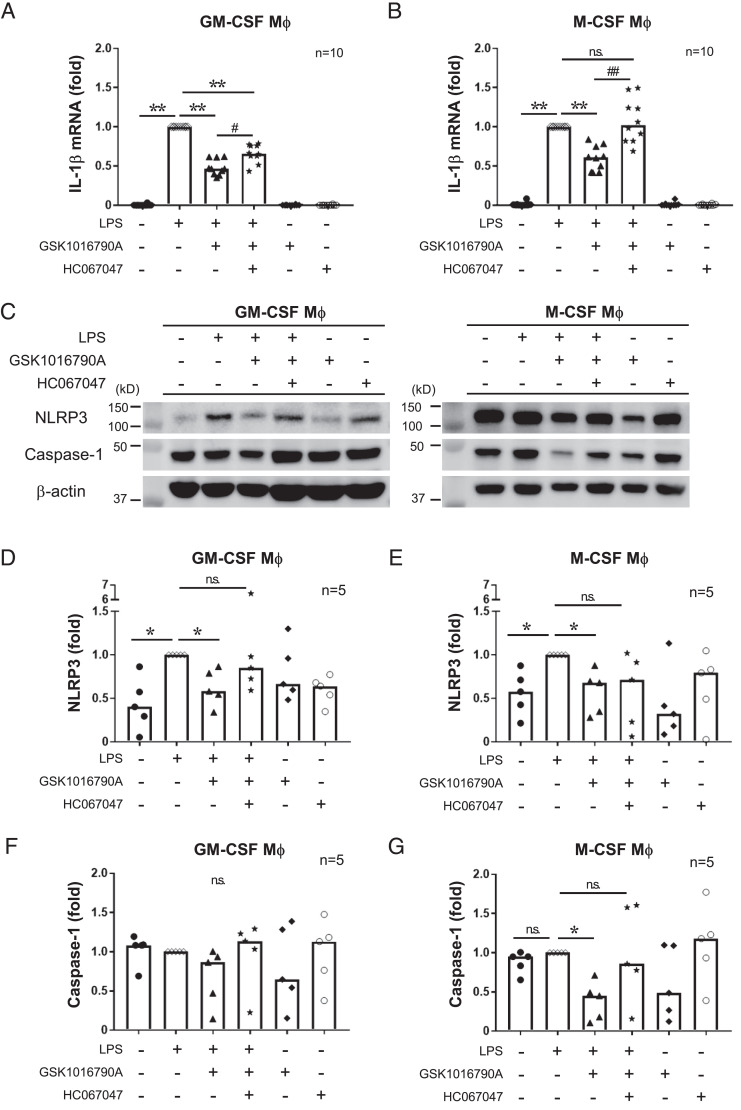
Activating TRPV4 suppresses mRNA expression of IL-1β and NLRP3 in macrophages. (**A** and **B**) The mRNA expression level of IL-1β in GM-CSF or M-CSF macrophages (Mφ) measured by RT-qPCR. Cells were stimulated for 6 h with 10 ng/ml LPS and 10 µM GSK1016790A following a 30-min 30-µM HC067047 pretreatment. IL-1β values were normalized to GAPDH mRNA expression and the IL-1β/GAPDH values normalized to the LPS-alone group. Bar graph shows median. **p < 0.01, ^#^*p* < 0.05, ^##^*p* < 0.01, Steel test; *n* = 10 donors. (**C**) Representative protein expression of NLRP3 (110 kDa), caspase-1 (48 kDa), and β-actin (40 kDa) in GM-CSF or M-CSF macrophages detected by Western blot. Cells were stimulated with 10 ng/ml LPS and 10 µM GSK1016790A for 6 h, 30 min after a 30-µM HC067047 pretreatment. (**D**–**G**) The band intensities of (C) were quantified using ImageJ. Values were normalized to β-actin intensity and the LPS-alone group. Bar graph shows median. *p < 0.05, Steel test; *n* = 5 donors.

Having demonstrated that TRPV4 activation suppressed the production of IL-1β, we next sought to further understand the mechanisms of how TRPV4 regulates IL-1β expression by first analyzing IL-1β mRNA expression by RT-qPCR. Among all three cell types, including GM-CSF macrophages, M-CSF macrophages, and primary monocytes, TRPV4 activation significantly decreased IL-1β mRNA expression, whereas pretreatment with the TRPV4 inhibitor HC067047 minimized the effect of GSK1016790A (Fig. 3A, 3B, Supplemental Fig. 1J). These results suggest that TRPV4 activation downregulates IL-1β mRNA expression. Next, we asked if TRPV4 activation regulates NLRP3 and procaspase-1 expression levels, because premature IL-1β protein is processed into mature IL-1β by inflammasomes composed of NLRP3 and caspase-1. We found that the expression of NLRP3 was decreased by TRPV4 activation in both GM-CSF and M-CSF macrophages (Fig. 3C–E). Interestingly, the physiological function of TRPV4 on procaspase-1 regulation was different between the two macrophage types; procaspase-1 levels were decreased in M-CSF macrophages in a TRPV4 activation-dependent manner. In contrast, procaspase-1 expression was not decreased in GM-CSF macrophages, although the cells were stimulated with GSK1016790A (Fig. 3F, 3G). These data suggest that TRPV4 activation suppresses not only IL-1β mRNA, but also NLRP3 expression, raising the possibility that the physiological role of TRPV4 in procaspase-1 regulation is different between GM-CSF and M-CSF macrophages.

### Activating TRPV4 suppresses activation of the NF-κB pathway

**FIGURE 4.**
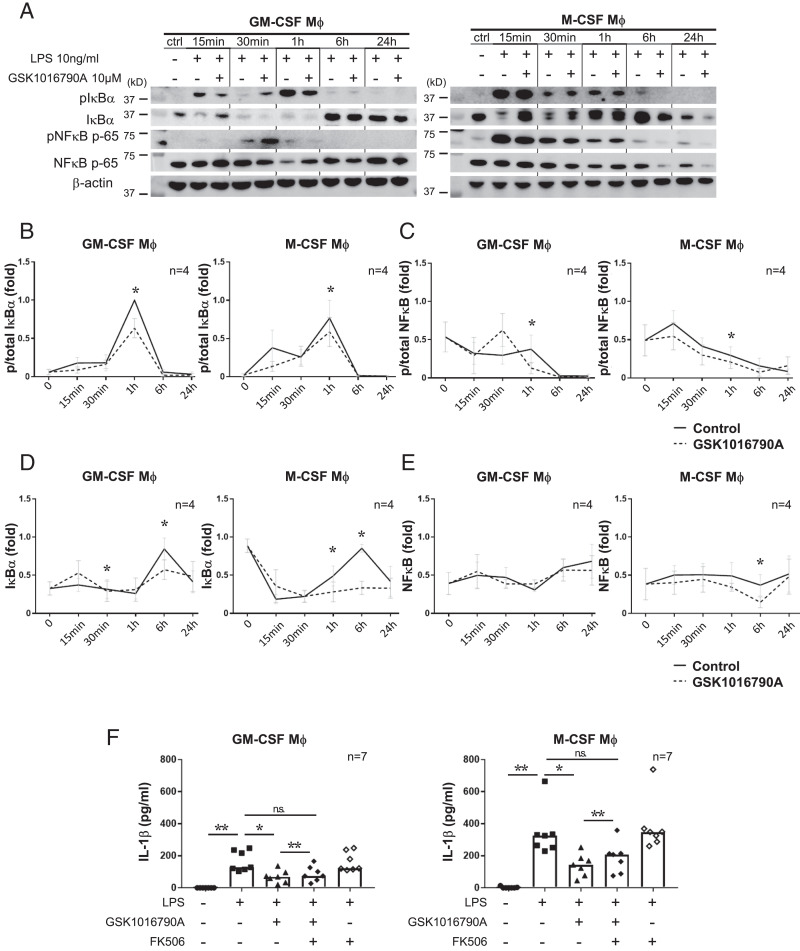
Activating TRPV4 suppresses NF-κB activity in macrophages. (**A**) Representative protein expression of phosphorylated and total IκBα (39 kDa), NF-κB (65 kDa), and β-actin (40 kDa) in GM-CSF or M-CSF macrophages (Mφ). Cells were costimulated with 10 ng/ml LPS and 10 µM GSK1016790A for 15 min, 30 min, 1 h, 6 h, and 24 h. (**B**–**E**) Band intensities in (A) were quantified using ImageJ software. p-IκBα/total IκBα (B), p–NF-κB/total NF-κB (C), total IκBα/β-actin (D), and total NF-κB/β-actin (E) are shown. All values in each group were normalized to the maximum value. The line graph shows the mean ± SEM. *p < 0.05, Wilcoxon signed-rank test, one-sided; *n* = 4 donors. (**F**) IL-1β production in GM-CSF or M-CSF macrophages measured by ELISA. Cells were costimulated with 10 ng/ml LPS and 10 µM GSK1016790A for 6 h after a 30-min pretreatment with 1 nM FK506. Bar graph shows the median. *p < 0.05, **p < 0.01, Steel test and paired *t* test (LPS plus GSK1016790A vs. LPS plus GSK1016790A plus FK506); *n* = 7 donors.

IL-1β and NLRP3 expression are regulated by NF-κB activity, and this transcriptional system is tightly regulated by cross-talk with several pathways, including JNK and cAMP-CREB signaling (29–31). To ask if TRPV4 activation modifies one of these signaling pathways or a transcription factor, we performed a luciferase assay with HEK293 cells constitutively expressing human TLR4, MD2, and CD14 (32, 33). Interestingly, the overexpression of TRPV4 highly activated all transcriptional activities (Supplemental Fig. 2A–F). Among the six reporters, NF-κB, NFAT, PU.1, and IRF5 activation in the combination GSK1016790A and LPS-treated group changed significantly compared with treatment with LPS alone. Cotreating with LPS and GSK1016790A activated NFAT, PU.1, and IRF5 compared with the LPS-stimulated group, whereas NF-κB activation was significantly decreased (Supplemental Fig. 2A–F). These results suggest that TRPV4 activation suppressed NF-κB signaling while activating NFAT, PU.1, and IRF5. To further understand the regulation mechanisms by which TRPV4 activation downregulates NF-κB signaling, we examined the expression and phosphorylation level of NF-κB components with time dependency in macrophages. Notably, the expression pattern of each component was different between GM-CSF and M-CSF macrophages. IκBα and p65 were activated earlier in M-CSF macrophages, and these activation peaks occurred 15 min after LPS stimulation (Fig. 4A–E). In contrast to M-CSF macrophages, GM-CSF macrophages activated these components later, with the activation peak of these components occurring 30 min or later. In both macrophages, TRPV4 activation significantly decreased the activation of NF-κB signaling components, which suggests that TRPV4 inhibits NF-κB signaling by decreasing the phosphorylation of IκBα and p-65.

We demonstrated that TRPV4 activation decreased the phosphorylation of NF-κB components, whereas JNK-NFAT signaling was activated (Fig. 4A–E, Supplemental Fig. 2A–F). As NFAT activation is regulated by a balance between phosphorylation and dephosphorylation, we hypothesized that TRPV4 may regulate kinase or phosphatase activity. Zaccor et al. (34) reported that TRPV4 activation induces Ca^2+^ influx, which activates calcineurin phosphatase. Therefore, we examined whether a specific inhibitor of calcineurin FK506 could inhibit the NFAT activity induced by TRPV4 activation. A luciferase assay suggested that calcineurin inhibition by FK506 significantly decreased GSK1016790A-induced NFAT activation (Supplemental Fig. 2G). Cotreating with FK506, GSK1016790A, and LPS did not change the IL-1β expression compared with LPS alone (Fig. 4F), which suggests that the TRPV4-calcineurin pathway regulates NF-κB, the NFAT pathway, and IL-1β expression.

### TRPV4 activation inhibits M1, but not M2, macrophage differentiation

**FIGURE 5.**
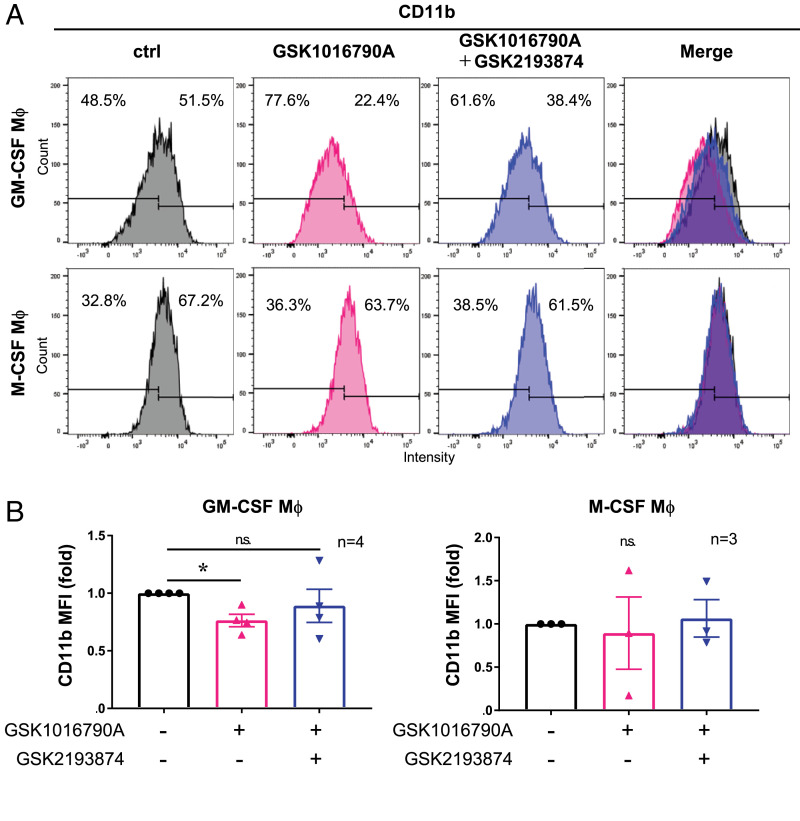
Activating TRPV4 suppresses cell differentiation from monocytes into GM-CSF, but not M-CSF, macrophages. Monocytes were differentiated into GM-CSF or M-CSF macrophages (Mφ) with or without 10 µM GSK1016790A or 1 µM GSK2193874 for 7 d. (**A**) CD11b expression in GM-CSF or M-CSF macrophages as measured by flow cytometry. (**B**) Mean fluorescence intensity (MFI) of CD11b in GM-CSF and M-CSF macrophages. MFI was quantified using FlowJo software. *n* = 4 donors in GM-CSF macrophages and *n* = 3 donors in M-CSF macrophages. The bar graph shows the mean values and the error bars indicate the SEM. *p < 0.05, Steel test.

Next, we investigated whether TRPV4 was involved in the differentiation of monocytes into macrophages. We cultured monocytes with GM-CSF or M-CSF cytokines and supplemented with the TRPV4 activator GSK1016790A for 7 d. To quantitatively analyze the effect of TRPV4 activation on macrophage differentiation, we performed flow cytometry to measure cell surface markers. Our data suggest that CD11b^high^ populations were decreased in GM-CSF macrophages cultured with GSK1016790A and that this effect was inhibited by GSK2193874, a TRPV4 antagonist. In contrast, the expression of CD11b in M-CSF macrophages did not change even after treatment with GSK1016790A or GSK2193874 (Fig. 5A, 5B, Supplemental Fig. 2H). Our flow cytometry data suggested that TRPV4 activation inhibited GM-CSF macrophage differentiation, but not M-CSF macrophage differentiation.

### M1 macrophages that do not express TRPV4 are increased in the dermis of AD

**FIGURE 6.**
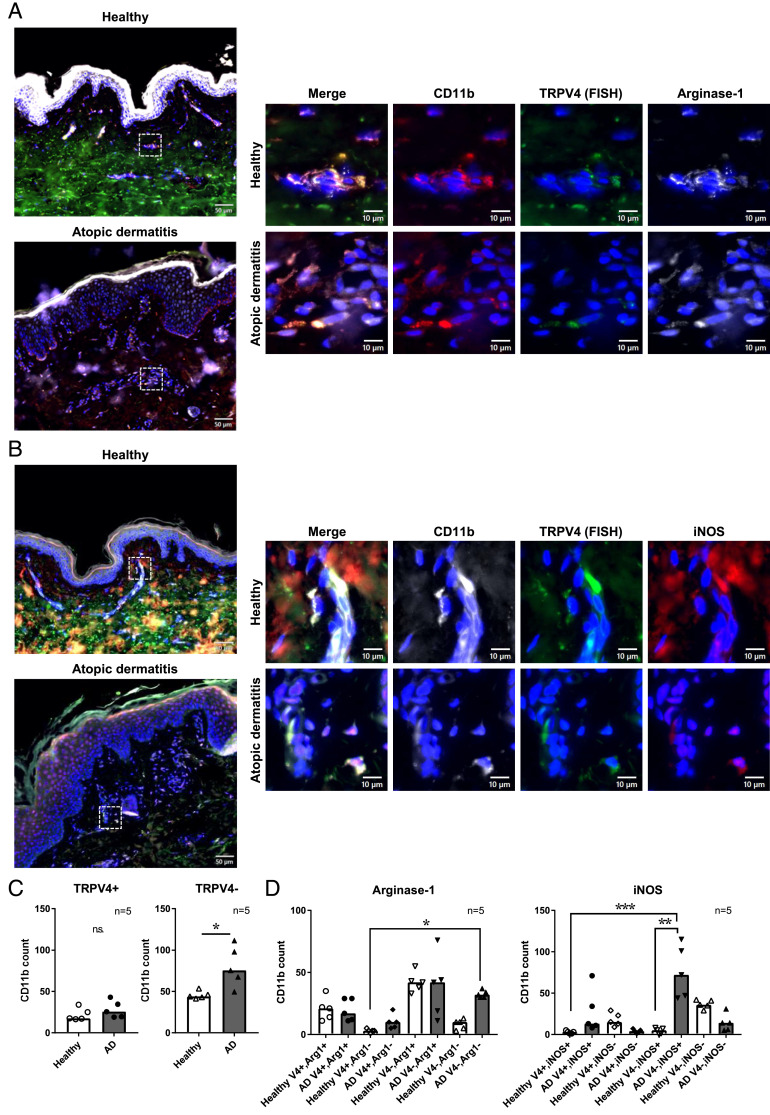
Downregulation of TRPV4 increases iNOS-positive macrophage presence during AD. (**A**) Representative fluorescent images of CD11b (red), TRPV4 (green; FISH), arginase-1 (white), and DAPI-stained nuclei in healthy and AD dermis (left; scale bars, 50 µm). The rectangular areas (white dashed line) were magnified and shown on the right (scale bars, 10 µm). (**B**) Representative fluorescent images of CD11b (white), TRPV4 (green, FISH), iNOS (red), and DAPI-stained nuclei in healthy and AD dermis (left; scale bars, 50 µm). The rectangular areas (white dashed line) were magnified and shown on the right (scale bars, 10 µm). (**C**) TRPV4 expression in dermal CD11b-positive macrophages in healthy and AD dermis. TRPV4-positive and TRPV4-negative populations in dermal macrophages were quantified. *p < 0.05, Welch *t* test; *n* = 5 donors. The number of CD11b-positive macrophages with each characteristic, arginase-1 (**D**) and iNOS (**E**). Five independent healthy volunteers and five independent AD skin specimens were examined. *p < 0.05, **p < 0.01, ***p < 0.001, Kruskal-Wallis test.

Having clarified that TRPV4 activation suppresses inflammatory responses in human macrophages and monocytes, we next investigated the relationship between TRPV4 expression and macrophage polarization markers in inflammatory skin diseases. We observed TRPV4 mRNA and macrophage marker proteins in human skin specimens of AD and psoriasis using a combination of FISH and fluorescent immunostaining. We observed that macrophages were primarily in the dermis, but not the epidermis, of all skin samples (Fig. 6A, 6B, Supplemental Figs. 3, 4A, 4B). In AD, TRPV4-negative macrophages were significantly increased compared with healthy skin (Fig. 6C, Supplemental Fig. 4A–E). In psoriasis, dermal macrophages rarely expressed TRPV4 mRNA, whereas the keratinocytes in these specimens did (Supplemental Fig. 3B, 3C). To investigate the physiological and pathological functions of TRPV4 in macrophages, we next investigated the relationship of M1 macrophage differentiation and TRPV4 expression in AD. We stained human skin with the M1 macrophage marker iNOS, M2 macrophage marker arginase-1, and pan-macrophage markers CD11b and CD68 as well as TRPV4 mRNA by FISH. When we investigated the M1/M2 macrophage population and TRPV4 expression patterns, the count of TRPV4-negative, iNOS-positive macrophages was significantly increased in AD compared with healthy skin (Fig. 6B, 6E, Supplemental Fig. 4B, 4D, 4G), whereas the percentage of arginase-1–positive macrophages was not changed by TRPV4 expression (Fig. 6A, 6D, Supplemental Fig. 4A, 4C, 4F). As our data suggested that iNOS-positive, TRPV4-negative macrophages were increased in AD, we hypothesized that TRPV4 in dermal macrophages polarized to M2 but not M1 macrophages.

In this study, we highlight that TRPV4 activation inhibits inflammatory cytokine expression, NF-κB signaling, and M1 polarization in human macrophages. Although we have not clarified the physiological function of TRPV4 in macrophages during skin homeostasis, our data raise the possibility that TRPV4 works as an inflammation suppressor in human macrophages.

## Discussion

In this study, we showed that TRPV4 activation suppressed IL-1β production in human macrophages and monocytes by inhibiting the NF-κB pathway (Figs. 1–4, Supplemental Figs. 1, 2). The calcineurin inhibitor FK506 reduced NFAT activation and rescued IL-1β reduction induced by the TRPV4 agonist in macrophages, suggesting that IL-1β production is also regulated by NFAT signaling and that the TRPV4-calcineurin axis activates the NFAT pathway while inhibiting NF-κB signaling and cytokine production. We also found that the TRPV4 functions to suppress GM-CSF macrophage polarization (Figs. 5, 6, Supplemental Figs. 2–4). TRPV4-negative-/iNOS-positive macrophages were significantly increased in AD compared with healthy skin dermis, which suggests a correlation between TRPV4 expression and M1 macrophage differentiation in human skin. Our results highlight the TRPV4 function as an inflammatory regulator in human macrophages.

A component of Gram-negative bacteria, LPS is widely accepted as a standard TLR4 ligand. The activation of TLR4 initiates the NF-κB and MAPK signaling pathway, which induces the production of proinflammatory cytokines. Because the alteration of skin microbiota is widely observed in several skin diseases (35) and this alteration may be the cause of such diseases, we focused on TLR4 signals in macrophages. It is well known that *Staphylococcus aureus* infections worsen AD symptoms, and cutaneous immune systems are modulated by commensal microbiota alterations (36, 37). Takeuchi et al. (38) demonstrated that TNF-α expression was significantly diminished in TLR4 knockout mice compared with wild-type mice, despite stimulating mice with LTA derived from *S. aureus* membrane components. This finding strongly suggested that *S. aureus* infections activate TLR4 signaling and raise the possibility that TLR4 is activated in AD lesions. Further studies regarding TRPV4 and TLR4 signaling in vivo are required to address this question.

The mechanism by which TRPV4 suppresses cytokine production is still unclear. After we found that TRPV4 activation decreased the availability of phosphorylated NF-κB components, we hypothesized that TRPV4 signaling may activate a phosphatase that targets NF-κB components. It has been reported that TRPV4 directly activates calcineurin phosphatase (34, 39, 40). These reports led us to treat macrophages with FK506, a calcineurin inhibitor. FK506 showed a partial but significant effect on GSK1016790A treatment in IL-1β production (Fig. 4F), which suggests the cross-talk of these signaling pathways. Previous work has shown that a Ca^2+^ influx through TRPC1 channels increases NF-κB expression as well as the expression of A20, a protein that negatively feeds back on NF-κB (41). In our data, we found that the expression of phosphorylated NF-κB induced by a 30-min treatment with both LPS and GSK1016790A was higher than that of LPS stimulation alone (Fig. 3A, 3C). Our finding raised the possibility that TRPV4 activation induced the negative feedback of NF-κB signaling, which suppressed the production of cytokines like IL-1β.

The regulation of IL-1β expression was TRPV4 activation–dependent, but the regulation of the expression of other cytokines, including IL-1α, IL-6, IL-10, and TNF-α, was not; we did not find any effect of the TRPV4 inhibitor on cytokine production (Supplemental Fig. 1E–G). Unlike IL-10, IL-6, and TNF-α (42), IL-1β requires the NLRP3 inflammasome for its maturation, suggesting that TRPV4 regulates the NLRP3 pathway. We also examined the interesting possibility that TRPV4 activation had different effects on each transcription factor. When we activated TRPV4 with GSK1016790A during LPS stimulation, NF-κB luciferase activity significantly decreased, whereas the activity of NFAT, PU.1, and IRF5 increased (Supplemental Fig. 2). Treatment with HC067047 during LPS stimulation did not change NF-κB activity compared with LPS stimulation alone, although NFAT activity tended to decrease (data not shown). Our data suggest that NFAT regulation is sensitive to Ca^2+^ concentration, and cytokine production may have been affected by the rigorous balance of transcription factors, affecting other cytokine levels.

The physiological roles of M1/M2 macrophages remain unclear. Unexpectedly, we found that M2 anti-inflammatory M-CSF macrophages expressed high levels of NLRP3 compared with M1 GM-CSF inflammatory macrophages (Fig. 3C). We also found that NF-κB components were activated earlier in M-CSF macrophages than in GM-CSF macrophages (Fig. 4A). It is widely accepted that M2 macrophages are dominant in the healthy dermis (5) (Fig. 6, Supplemental Fig. 4). Our findings raise an interesting possibility that dermal M2 macrophages react more quickly than M1 macrophages, which allows the maintenance of tissue homeostasis. Although NLRP3 expression in M1 GM-CSF macrophages was less than M-CSF macrophages, GM-CSF macrophages showed a similar expression level of inflammatory cytokines, including IL-1β (Fig. 2). In contrast to inflammatory cytokines, IL-10 anti-inflammatory cytokines were low compared with M-CSF macrophages (Fig. 2C, 2D). Our data still do not provide an insight into the physiological functions of each macrophage population; however, we hypothesize that M1 macrophages can accelerate inflammation in tissues because they do not weaken inflammatory responses. It has been reported that NLRP3 promotes macrophage polarization to the M2 by increasing IL-4 production (43). Although NLRP3 regulation mechanisms remain unclear, our findings support our hypothesis that TRPV4 regulates NLRP3 and M2 polarization.

In general, the activation of temperature-sensitive TRP channels at the optimum temperature was thought to be partial, and the effects of ligands, hypotonic conditions, and agonists are usually exerted even at the optimal temperature (18). Therefore, we hypothesized that TRPV4 is not fully activated at physiological temperature without a ligand/agonist. Interestingly, some recent articles suggest a relationship between climatologic factors and AD. Low temperature and low humidity may lead to skin barrier dysfunction and increase the risk of dermatitis (44); however, the relationship between immune responses, temperature, and skin diseases remains unknown. Investigations of the local temperature in AD lesions will be pursued in future work. 5′, 6’-Epoxyeicosatrienoic acid (EET), the cytochrome P450-mediated metabolite of arachidonic acid (45, 46), has been identified as an endogenous ligand for TRPV4. Many reports suggest that macrophage function is regulated by the balance of fatty acid metabolism (47), and it is also reported that epoxy fatty acids have anti-inflammatory and tissue-protective effects (48, 49). CYP2J2, a metabolic enzyme of EET, is expressed in human monocytes and macrophages (50, 51), and EET regulates both the balance of fatty acid metabolism and TRPV4 activation. As the expression of CYP2J2 in AD decreases compared with healthy tissue (52), it is possible that EET expression in AD is low, which may result in TRPV4 inactivation and hyperimmunoreactivity. The expression levels of EET and CYP2J2 in skin diseases other than AD are not clear, so further investigation is required.

In this study, we found that TRPV4 expression is decreased in dermal macrophages during AD and psoriasis. These inflammatory skin diseases also have differing cytokine expression patterns. Therefore, we hypothesized that a specific cytokine may change TRPV4 expression. In AD, a Th2-dominant response is overproduced and prevents the transition to a Th1-dominant response (12). We note that one of the study limitations is that we have not yet identified the regulation mechanisms of TRPV4 expression/activation in AD. Future work focusing on the regulation of TRPV4 expression by cytokine stimulation should be performed. Although we have not clarified the regulatory mechanism of TRPV4 expression and activation in diseases, we hypothesize that the change in expression of TRPV4 leads to the dysregulation of homeostasis. The dysregulation of IL-1 family signaling has been observed in several skin diseases and linked to the pathology of psoriasis and AD (11, 53, 54). Our observation that the activation of TRPV4 in monocytes and macrophages suppressed the production of inflammatory cytokines, such as IL-1β, as well as differentiation into inflammatory M1 macrophages, led us to hypothesize that this anti-inflammatory effect may contribute to the prevention of excessive inflammation. Furthermore, TRPV4 activation suppressed IL-10 production in M-CSF macrophages (Fig. 2D), which may prevent Th2-dominant responses. From our findings, we believe that TRPV4 activators are a potential new therapeutic for AD. Further research using AD animal models is required to explore this possibility.

## Supplementary Material

Supplemental Figure 1 (PDF)

## References

[1] Metchnikoff E. 1892. Lectures on the Comparative Pathology of Inflammation. Kegan Paul, London.

[2] Mosser D. M., J. P. Edwards. 2008. Exploring the full spectrum of macrophage activation. [Published erratum appears in 2010 *Nat. Rev. Immunol.* 10: 460.] Nat. Rev. Immunol. 8: 958–969. 19029990 10.1038/nri2448 PMC2724991

[3] Mantovani A., A. Sica, S. Sozzani, P. Allavena, A. Vecchi, M. Locati. 2004. The chemokine system in diverse forms of macrophage activation and polarization. Trends Immunol. 25: 677–686. 15530839 10.1016/j.it.2004.09.015

[4] Arango Duque G., A. Descoteaux. 2014. Macrophage cytokines: involvement in immunity and infectious diseases. Front. Immunol. 5: 491. 25339958 10.3389/fimmu.2014.00491 PMC4188125

[5] Italiani P., D. Boraschi. 2014. From monocytes to M1/M2 macrophages: phenotypical vs. functional differentiation. Front. Immunol. 5: 514. 25368618 10.3389/fimmu.2014.00514 PMC4201108

[6] Davies L. C., S. J. Jenkins, J. E. Allen, P. R. Taylor. 2013. Tissue-resident macrophages. Nat. Immunol. 14: 986–995. 24048120 10.1038/ni.2705 PMC4045180

[7] Natsuaki Y., G. Egawa, S. Nakamizo, S. Ono, S. Hanakawa, T. Okada, N. Kusuba, A. Otsuka, A. Kitoh, T. Honda, 2014. Perivascular leukocyte clusters are essential for efficient activation of effector T cells in the skin. Nat. Immunol. 15: 1064–1069. 25240383 10.1038/ni.2992

[8] Egawa M., K. Mukai, S. Yoshikawa, M. Iki, N. Mukaida, Y. Kawano, Y. Minegishi, H. Karasuyama. 2013. Inflammatory monocytes recruited to allergic skin acquire an anti-inflammatory M2 phenotype via basophil-derived interleukin-4. Immunity 38: 570–580. 23434060 10.1016/j.immuni.2012.11.014

[9] Willis C. M., S. Shaw, O. De Lacharrière, M. Baverel, L. Reiche, R. Jourdain, P. Bastien, J. D. Wilkinson. 2001. Sensitive skin: an epidemiological study. Br. J. Dermatol. 145: 258–263. 11531788 10.1046/j.1365-2133.2001.04343.x

[10] Pastore S., E. Fanales-Belasio, C. Albanesi, L. M. Chinni, A. Giannetti, G. Girolomoni. 1997. Granulocyte macrophage colony-stimulating factor is overproduced by keratinocytes in atopic dermatitis. Implications for sustained dendritic cell activation in the skin. J. Clin. Invest. 99: 3009–3017. 9185525 10.1172/JCI119496 PMC508153

[11] Martin P., J. D. Goldstein, L. Mermoud, A. Diaz-Barreiro, G. Palmer. 2021. IL-1 family antagonists in mouse and human skin inflammation. Front. Immunol. 12: 652846. 33796114 10.3389/fimmu.2021.652846 PMC8009184

[12] Ohmen J. D., J. M. Hanifin, B. J. Nickoloff, T. H. Rea, R. Wyzykowski, J. Kim, D. Jullien, T. McHugh, A. S. Nassif, S. C. Chan, 1995. Overexpression of IL-10 in atopic dermatitis. Contrasting cytokine patterns with delayed-type hypersensitivity reactions. J. Immunol. 154: 1956–1963. 7836775

[13] Caterina M. J., M. A. Schumacher, M. Tominaga, T. A. Rosen, J. D. Levine, D. Julius. 1997. The capsaicin receptor: a heat-activated ion channel in the pain pathway. Nature 389: 816–824. 9349813 10.1038/39807

[14] Nilius B. 2007. TRP channels in disease. Biochim. Biophys. Acta Mol. Basis Dis. 1772: 805–812. 10.1016/j.bbadis.2007.02.002 17368864

[15] Yue L., H. Xu. 2021. TRP channels in health and disease at a glance. J. Cell Sci. 134: jcs258372. 34254641 10.1242/jcs.258372 PMC8358089

[16] Lehen′kyi V., N. Prevarskaya. 2011. Chapter 17: Study of TRP channels in cancer cells. In TRP Channels. M. X. Zhu, ed. CRC Press/Taylor & Francis, Boca Raton, FL, p. https://www.ncbi.nlm.nih.gov/books/NBK92828/. 22593971

[17] Prevarskaya N., L. Zhang, G. Barritt. 2007. TRP channels in cancer. Biochim. Biophys. Acta Mol. Basis Dis. 1772: 937–946. 10.1016/j.bbadis.2007.05.006 17616360

[18] Güler A. D., H. Lee, T. Iida, I. Shimizu, M. Tominaga, M. Caterina. 2002. Heat-evoked activation of the ion channel, TRPV4. J. Neurosci. 22: 6408–6414. 12151520 10.1523/JNEUROSCI.22-15-06408.2002 PMC6758176

[19] Sokabe T., T. Fukumi-Tominaga, S. Yonemura, A. Mizuno, M. Tominaga. 2010. The TRPV4 channel contributes to intercellular junction formation in keratinocytes. J. Biol. Chem. 285: 18749–18758. 20413591 10.1074/jbc.M110.103606 PMC2881798

[20] Kida N., T. Sokabe, M. Kashio, K. Haruna, Y. Mizuno, Y. Suga, K. Nishikawa, A. Kanamaru, M. Hongo, A. Oba, M. Tominaga. 2012. Importance of transient receptor potential vanilloid 4 (TRPV4) in epidermal barrier function in human skin keratinocytes. Pflugers Arch. 463: 715–725. 22374181 10.1007/s00424-012-1081-3

[21] Fu S., H. Meng, S. Inamdar, B. Das, H. Gupta, W. Wang, C. L. Thompson, M. M. Knight. 2021. Activation of TRPV4 by mechanical, osmotic or pharmaceutical stimulation is anti-inflammatory blocking IL-1β mediated articular cartilage matrix destruction. Osteoarthritis Cartilage 29: 89–99. 33395574 10.1016/j.joca.2020.08.002 PMC7799379

[22] Scheraga R. G., B. D. Southern, L. M. Grove, M. A. Olman. 2017. The role of transient receptor potential vanilloid 4 in pulmonary inflammatory diseases. Front. Immunol. 8: 503. 28523001 10.3389/fimmu.2017.00503 PMC5415870

[23] Scheraga R. G., S. Abraham, K. A. Niese, B. D. Southern, L. M. Grove, R. D. Hite, C. McDonald, T. A. Hamilton, M. A. Olman. 2016. TRPV4 mechanosensitive ion channel regulates lipopolysaccharide-stimulated macrophage phagocytosis. J. Immunol. 196: 428–436. 26597012 10.4049/jimmunol.1501688 PMC4684994

[24] Lacey D. C., A. Achuthan, A. J. Fleetwood, H. Dinh, J. Roiniotis, G. M. Scholz, M. W. Chang, S. K. Beckman, A. D. Cook, J. A. Hamilton. 2012. Defining GM-CSF- and macrophage-CSF-dependent macrophage responses by in vitro models. J. Immunol. 188: 5752–5765. 22547697 10.4049/jimmunol.1103426

[25] Fujita F., K. Uchida, M. Takaishi, T. Sokabe, M. Tominaga. 2013. Ambient temperature affects the temperature threshold for TRPM8 activation through interaction of phosphatidylinositol 4,5-bisphosphate. J. Neurosci. 33: 6154–6159. 23554496 10.1523/JNEUROSCI.5672-12.2013 PMC6618937

[26] Takaishi M., K. Uchida, F. Fujita, M. Tominaga. 2014. Inhibitory effects of monoterpenes on human TRPA1 and the structural basis of their activity. J. Physiol. Sci. 64: 47–57. 24122170 10.1007/s12576-013-0289-0 PMC3889502

[27] Everaerts W., X. Zhen, D. Ghosh, J. Vriens, T. Gevaert, J. P. Gilbert, N. J. Hayward, C. R. McNamara, F. Xue, M. M. Moran, 2010. Inhibition of the cation channel TRPV4 improves bladder function in mice and rats with cyclophosphamide-induced cystitis. Proc. Natl. Acad. Sci. USA 107: 19084–19089. 20956320 10.1073/pnas.1005333107 PMC2973867

[28] Thorneloe K. S., M. Cheung, W. Bao, H. Alsaid, S. Lenhard, M.-Y. Jian, M. Costell, K. Maniscalco-Hauk, J. A. Krawiec, A. Olzinski, 2012. An orally active TRPV4 channel blocker prevents and resolves pulmonary edema induced by heart failure. Sci. Transl. Med. 4: 159ra148. 10.1126/scitranslmed.3004276 23136043

[29] Boaru S. G., E. Borkham-Kamphorst, E. Van de Leur, E. Lehnen, C. Liedtke, R. Weiskirchen. 2015. NLRP3 inflammasome expression is driven by NF-κB in cultured hepatocytes. Biochem. Biophys. Res. Commun. 458: 700–706. 25686493 10.1016/j.bbrc.2015.02.029

[30] Nakano H. 2004. Signaling crosstalk between NF-kappaB and JNK. Trends Immunol. 25: 402–405. 15275637 10.1016/j.it.2004.05.007

[31] Wen A. Y., K. M. Sakamoto, L. S. Miller. 2010. The role of the transcription factor CREB in immune function. J. Immunol. 185: 6413–6419. 21084670 10.4049/jimmunol.1001829 PMC5519339

[32] Zheng M., A. Ambesi, P. J. McKeown-Longo. 2020. Role of TLR4 receptor complex in the regulation of the innate immune response by fibronectin. Cells 9: 216. 31952223 10.3390/cells9010216 PMC7017243

[33] Takahashi Y., M. E. Cueno, N. Kamio, T. Iinuma, Y. Hasegawa, K. Imai. 2022. *Porphyromonas gingivalis* Mfa1 fimbria putatively binds to TLR2 and induces both IL-6 and IL-8 production in human bronchial epithelial cells. Biochem. Biophys. Res. Commun. 589: 35–40. 34891039 10.1016/j.bbrc.2021.12.003

[34] Zaccor N. W., C. J. Sumner, S. H. Snyder. 2020. The nonselective cation channel TRPV4 inhibits angiotensin II receptors. J. Biol. Chem. 295: 9986–9997. 32493776 10.1074/jbc.RA120.014325 PMC7380189

[35] Grice E. A., J. A. Segre. 2011. The skin microbiome. [Published erratum appears in 2011 *Nat. Rev. Microbiol.* 9: 626.] Nat. Rev. Microbiol. 9: 244–253. 21407241 10.1038/nrmicro2537 PMC3535073

[36] Kobayashi T., M. Glatz, K. Horiuchi, H. Kawasaki, H. Akiyama, D. H. Kaplan, H. H. Kong, M. Amagai, K. Nagao. 2015. Dysbiosis and *Staphylococcus aureus* colonization drives inflammation in atopic dermatitis. Immunity 42: 756–766. 25902485 10.1016/j.immuni.2015.03.014 PMC4407815

[37] Ogonowska P., Y. Gilaberte, W. Barańska-Rybak, J. Nakonieczna. 2021. Colonization with *Staphylococcus aureus* in atopic dermatitis patients: attempts to reveal the unknown. Front. Microbiol. 11: 567090. 33505363 10.3389/fmicb.2020.567090 PMC7830525

[38] Takeuchi O., K. Hoshino, T. Kawai, H. Sanjo, H. Takada, T. Ogawa, K. Takeda, S. Akira. 1999. Differential roles of TLR2 and TLR4 in recognition of gram-negative and gram-positive bacterial cell wall components. Immunity 11: 443–451. 10549626 10.1016/s1074-7613(00)80119-3

[39] Masuyama R., J. Vriens, T. Voets, Y. Karashima, G. Owsianik, R. Vennekens, L. Lieben, S. Torrekens, K. Moermans, A. Vanden Bosch, 2008. TRPV4-mediated calcium influx regulates terminal differentiation of osteoclasts. Cell Metab. 8: 257–265. 18762026 10.1016/j.cmet.2008.08.002

[40] Zhao L., M. N. Sullivan, M. Chase, A. L. Gonzales, S. Earley. 2014. Calcineurin/nuclear factor of activated T cells-coupled vanilliod transient receptor potential channel 4 ca2+ sparklets stimulate airway smooth muscle cell proliferation. Am. J. Respir. Cell Mol. Biol. 50: 1064–1075. 24392954 10.1165/rcmb.2013-0416OC PMC4068915

[41] Thippegowda P. B., V. Singh, P. C. Sundivakkam, J. Xue, A. B. Malik, C. Tiruppathi. 2010. Ca2+ influx via TRPC channels induces NF-kappaB-dependent A20 expression to prevent thrombin-induced apoptosis in endothelial cells. Am. J. Physiol. Cell Physiol. 298: C656–C664. 20032510 10.1152/ajpcell.00456.2009 PMC2838565

[42] Sahin Ozkartal C., F. Aricioglu. 2013. Future directions of cytokine hypothesis in depression: “NLRP3 inflammasome. Klinik Psikofarmakol. Bülteni 23: 1.

[43] Liu Y., X. Gao, Y. Miao, Y. Wang, H. Wang, Z. Cheng, X. Wang, X. Jing, L. Jia, L. Dai, 2018. NLRP3 regulates macrophage M2 polarization through up-regulation of IL-4 in asthma. Biochem. J. 475: 1995–2008. 29626160 10.1042/BCJ20180086

[44] Engebretsen K. A., J. D. Johansen, S. Kezic, A. Linneberg, J. P. Thyssen. 2016. The effect of environmental humidity and temperature on skin barrier function and dermatitis. J. Eur. Acad. Dermatol. Venereol. 30: 223–249. 26449379 10.1111/jdv.13301

[45] Watanabe H., J. Vriens, J. Prenen, G. Droogmans, T. Voets, B. Nilius. 2003. Anandamide and arachidonic acid use epoxyeicosatrienoic acids to activate TRPV4 channels. Nature 424: 434–438. 12879072 10.1038/nature01807

[46] Berna-Erro A., M. Izquierdo-Serra, R. V. Sepúlveda, F. Rubio-Moscardo, P. Doñate-Macián, S. A. Serra, J. Carrillo-Garcia, A. Perálvarez-Marín, F. González-Nilo, J. M. Fernández-Fernández, M. A. Valverde. 2017. Structural determinants of 5′,6′-epoxyeicosatrienoic acid binding to and activation of TRPV4 channel. Sci. Rep. 7: 10522. 28874838 10.1038/s41598-017-11274-1 PMC5585255

[47] Oishi Y., N. J. Spann, V. M. Link, E. D. Muse, T. Strid, C. Edillor, M. J. Kolar, T. Matsuzaka, S. Hayakawa, J. Tao, 2017. SREBP1 contributes to resolution of pro-inflammatory TLR4 signaling by reprogramming fatty acid metabolism. Cell Metab. 25: 412–427. 28041958 10.1016/j.cmet.2016.11.009 PMC5568699

[48] Thomson S. J., A. Askari, D. Bishop-Bailey. 2012. Anti-inflammatory effects of epoxyeicosatrienoic acids. Int. J. Vasc. Med. 2012: 605101. 22848834 10.1155/2012/605101 PMC3405717

[49] Li R., X. Xu, C. Chen, Y. Wang, A. Gruzdev, D. C. Zeldin, D. W. Wang. 2015. CYP2J2 attenuates metabolic dysfunction in diabetic mice by reducing hepatic inflammation via the PPARγ. Am. J. Physiol. Endocrinol. Metab. 308: E270–E282. 25389363 10.1152/ajpendo.00118.2014 PMC4329496

[50] Nakayama K., T. Nitto, T. Inoue, K. Node. 2008. Expression of the cytochrome P450 epoxygenase CYP2J2 in human monocytic leukocytes. Life Sci. 83: 339–345. 18675280 10.1016/j.lfs.2008.06.026

[51] Bystrom J., J. A. Wray, M. C. Sugden, M. J. Holness, K. E. Swales, T. D. Warner, M. L. Edin, D. C. Zeldin, D. W. Gilroy, D. Bishop-Bailey. 2011. Endogenous epoxygenases are modulators of monocyte/macrophage activity. PLoS One 6: e26591. 22028915 10.1371/journal.pone.0026591 PMC3197524

[52] Ewald D. A., D. Malajian, J. G. Krueger, C. T. Workman, T. Wang, S. Tian, T. Litman, E. Guttman-Yassky, M. Suárez-Fariñas. 2015. Meta-analysis derived atopic dermatitis (MADAD) transcriptome defines a robust AD signature highlighting the involvement of atherosclerosis and lipid metabolism pathways. BMC Med. Genomics 8: 60. 26459294 10.1186/s12920-015-0133-x PMC4603338

[53] Matejuk A. 2018. Skin immunity. Arch. Immunol. Ther. Exp. (Warsz.) 66: 45–54. 28623375 10.1007/s00005-017-0477-3 PMC5767194

[54] Pasparakis M., I. Haase, F. O. Nestle. 2014. Mechanisms regulating skin immunity and inflammation. Nat. Rev. Immunol. 14: 289–301. 24722477 10.1038/nri3646

