# Supplementary material for: Anti-Inflammatory Role of TRPV4 in Human Macrophages
Source: Immunohorizons. 2023 Jan 16;7(1):81–96. doi: 10.4049/immunohorizons.2200100 (PMC10563396; doi:10.4049/immunohorizons.2200100)
Supplement: Supplemental Figure 1 (PDF) [file IH_2200100_Supplemental_1.pdf]

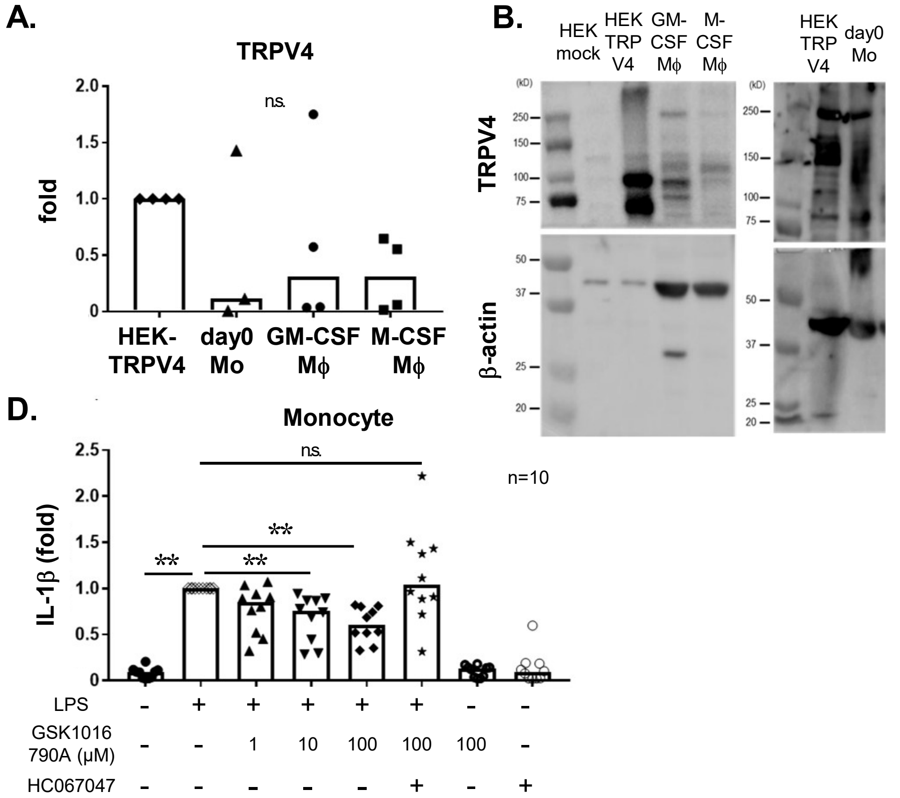

C. OOR< low high OOR>

	ctrl		LPS	
pg/ml	-	GSK1	-	GSK1
E-Selectin	4202.5	3508.33	5762.08	4786.47
GM-CSF	OOR <	OOR <	OOR <	OOR <
IFN-α	3.55	1.54	7.03	3.67
IFN-γ	2.62	1.51	13.11	4.83
IL-1α	61.29	54.88	107.61	93.66
IL-1β	22.71	24.18	125.57	105.06
IL-10	OOR <	OOR <	120.92	OOR <
IL-12p70	1.76	1.76	5.77	2.44
IL-13	48.19	56.58	177.01	80.06
IL-17a	27.32	23.78	34.61	30.27
IL-4	198.38	100.43	242.38	198.38
IL-6	899.5	669	2307.22	1123.71
IL-8	OOR >	OOR >	OOR >	OOR >
IP-10	3.82	3.03	6.56	4.38
MCP-1	98.36	29.09	164.07	39.14
MIP-1α	385.92	316.56	OOR >	1162.39
MIP-1β	4541.63	1056.87	18763.65	6076.39
P-Selectin	10640.49	10973.95	11307.17	13139.17
sICAM-1	12866.45	8949.84	14910.03	13503.92
TNF-α	25.23	15.21	54.24	35.96

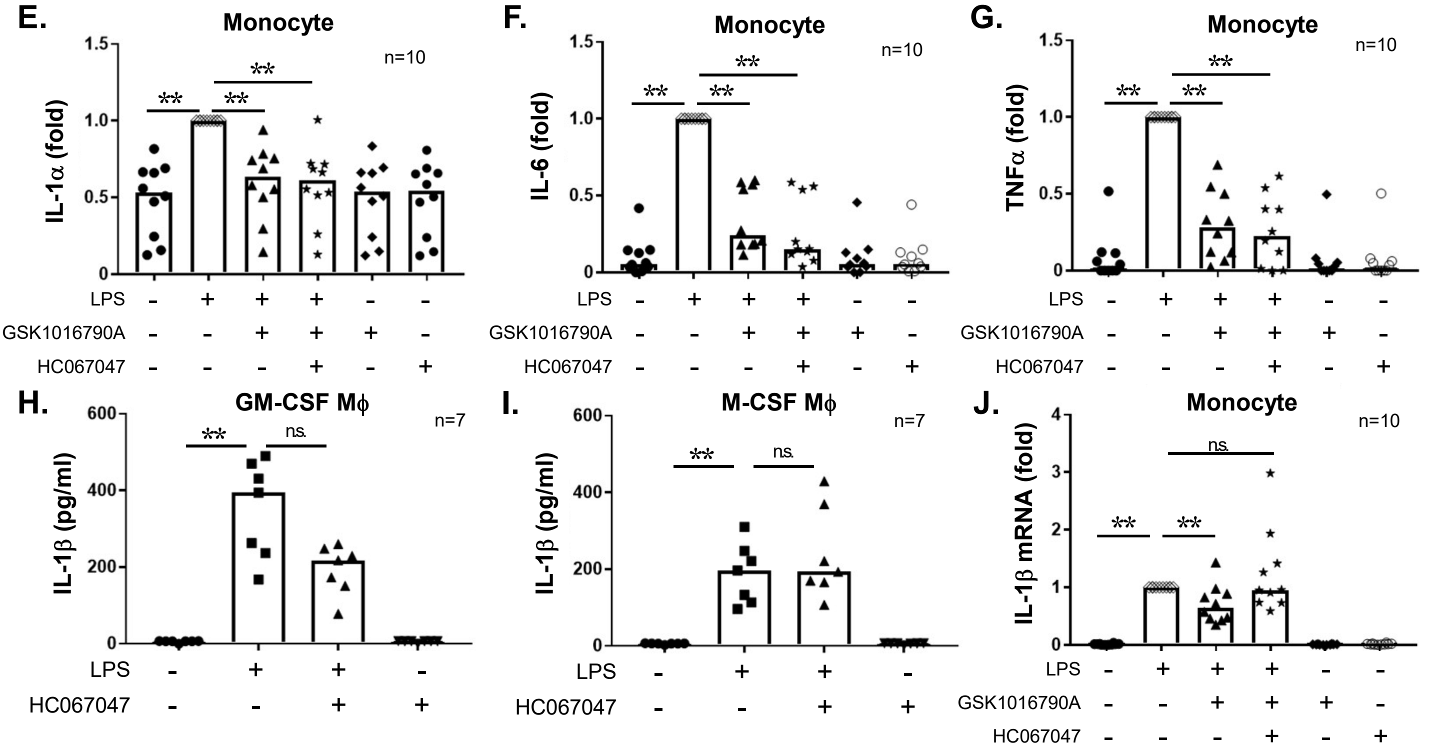

Figure S1. Activating TRPV4 suppresses the production of Th1 cytokines in monocytes.
(A) Quantification of TRPV4 expression in monocytes and macrophages. The band intensity of Figure 1B was quantified using Image J software. The bar graph shows the median values. Monocytes: n=3 donors, macrophages: n=4 donors. One-way ANOVA statistical analysis was performed. **(B)** Full blot of Figure 1B. **(C)** Cytokine production in monocytes detected by multiplex ELISA. Raw values (pg/ml) and heatmap of cytokine levels were shown. Primary human monocytes were co-stimulated with 10 pg/ml LPS and 100 μM GSK1016790A for 6 hours. OOR> means out of range above. OOR< means out of range below. **(D-G)** Expression levels of **(D)** IL-1β, **(E)** IL-1α, **(F)** IL-6, and **(G)** TNFα in monocytes measured by ELISA. Monocytes were co-stimulated with 10 pg/ml LPS and 100 μM GSK1016790A for 6 hours, 30 min after pretreatment with 30 μM HC067047. All values were normalized to the LPS-alone group. The graph shows the median values. *, $p < 0.05$ and **, $p < 0.01$, Steel test, n=10 individual donors. **(H, I)** IL-1β expression in **(H)** GM-CSF macrophages and **(I)** M-CSF macrophages. Macrophages were stimulated with 10 ng/ml LPS for 6 hours. Thirty minutes before LPS treatment, the cells were treated with 30 μM HC067047. The graph shows the median values. **, $p < 0.01$, Steel test, n=7 individual donors. **(J)** IL-1β mRNA level in monocytes measured by RT-qPCR. Monocytes were co-stimulated for 6 hours with 10 pg/ml LPS and 100 μM GSK1016790A following a 30 minute pretreatment with 30 μM HC067047. The values were normalized to the LPS-alone group. The graph shows the median values. *, $p < 0.05$ and **, $p < 0.01$, Steel test, n=10 individual donors.

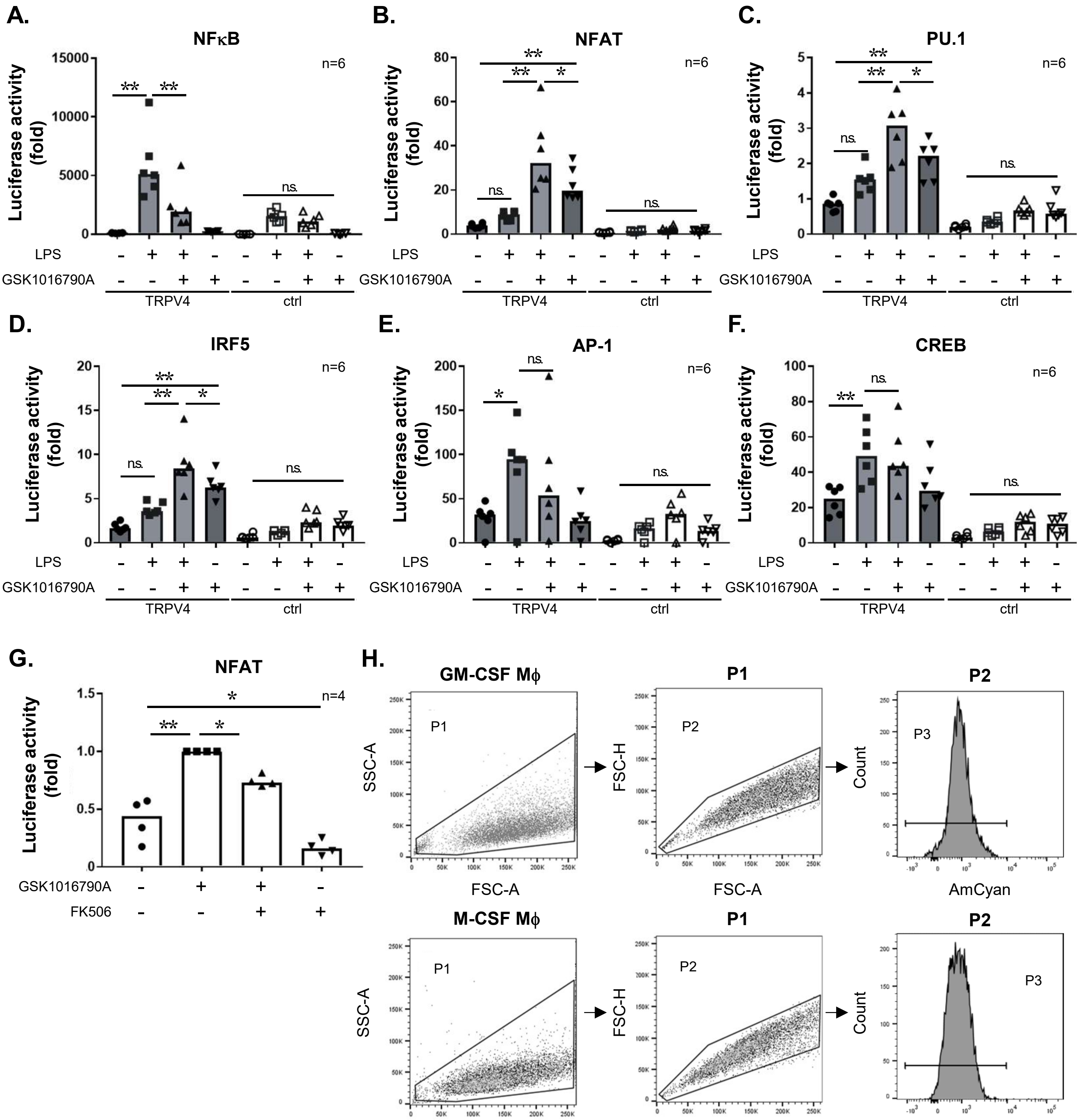

Figure S2. Activating TRPV4 regulates transcriptional PU.1 and IRF5 activity.
(A-F) Luciferase activity in 293/hTLR4-MD2-CD14 cells infected with the NanoLuc vector containing **(A)** NFκB, **(B)** NFAT, **(C)** PU.1, **(D)** IRF5, **(E)** AP-1, or **(F)** CREB response element. Firefly luciferase as the loading control and pcDNA3.1-TRPV4 (TRPV4) or the empty vector (ctrl) were co-transfected. Cells were stimulated with 1 µg/ml LPS and 50µM GSK1016790A for 24 hours. Values are relative to firefly luciferase activity. The bar graph shows the median values. *, $p < 0.05$ and **, $p < 0.01$, Tukey-Kramer test, n=6 individual experiments. **(G)** Luciferase activity in 293/hTLR4-MD2-CD14 cells transfected with the NFAT response element. Cells were overexpressed with pcDNA3.1-TRPV4 (TRPV4) then stimulated with 50 µM GSK1016790A and 1 nM FK506 for 24 hours. Values were normalized to firefly luciferase activity. The bar graph shows the median values. *, $p < 0.05$ and **, $p < 0.01$, Tukey-Kramer test, n=4 individual experiments. **(H)** Flow cytometry gating strategy for dead cell removal. Day-7 macrophages derived from monocytes were gated as P1 with FSC-A and SSC-A, then cells were gated as P2 with FSC-H and FSC-A. The AmCyan-negative population was gated as P3 to investigate CD11b expression.

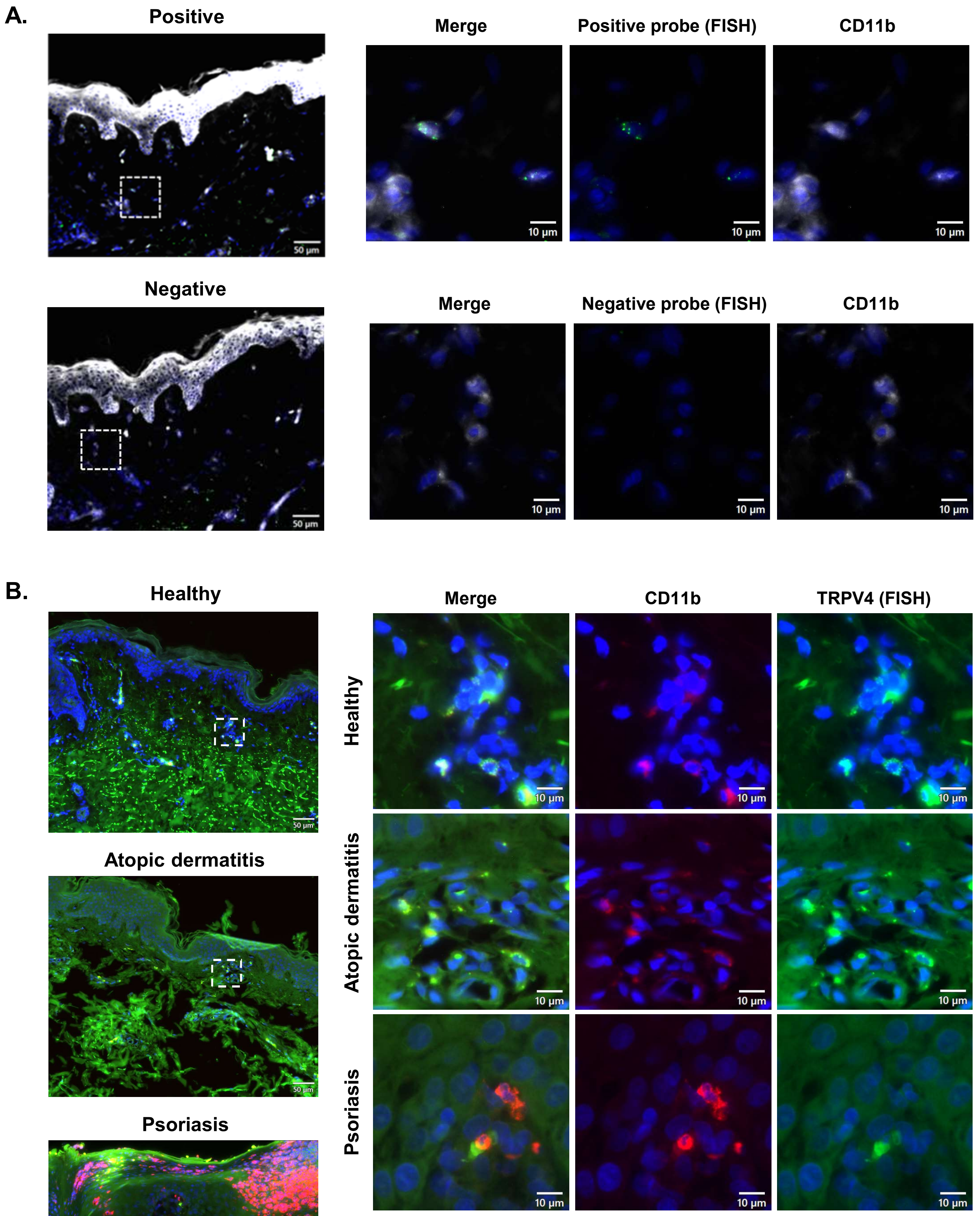

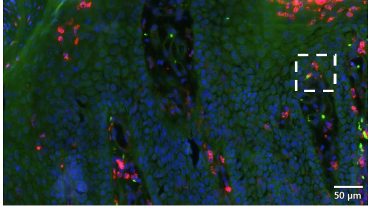

C.

	TRPV4	CD11b
Healthy	+++	++
Atopic dermatitis	+++	+++
Psoriasis	+	+++

Figure S3. Macrophage expression of TRPV4 in inflammatory skin diseases.
(A) Representative fluorescent images of healthy skin stained with positive or negative probes (green, FISH) with CD11b (white) and DAPI. The box with the dashed line indicates the enlarged area shown on the right. **(B)** Representative fluorescent images of healthy, atopic dermatitis, and psoriasis skin stained using CD11b (red), TRPV4 (green, FISH) and DAPI. The box (dashed line) indicates the enlarged area shown on the right. **(C)** Table of the characteristics of TRPV4 or CD11b expression patterns in each skin disease. n=2 donors.

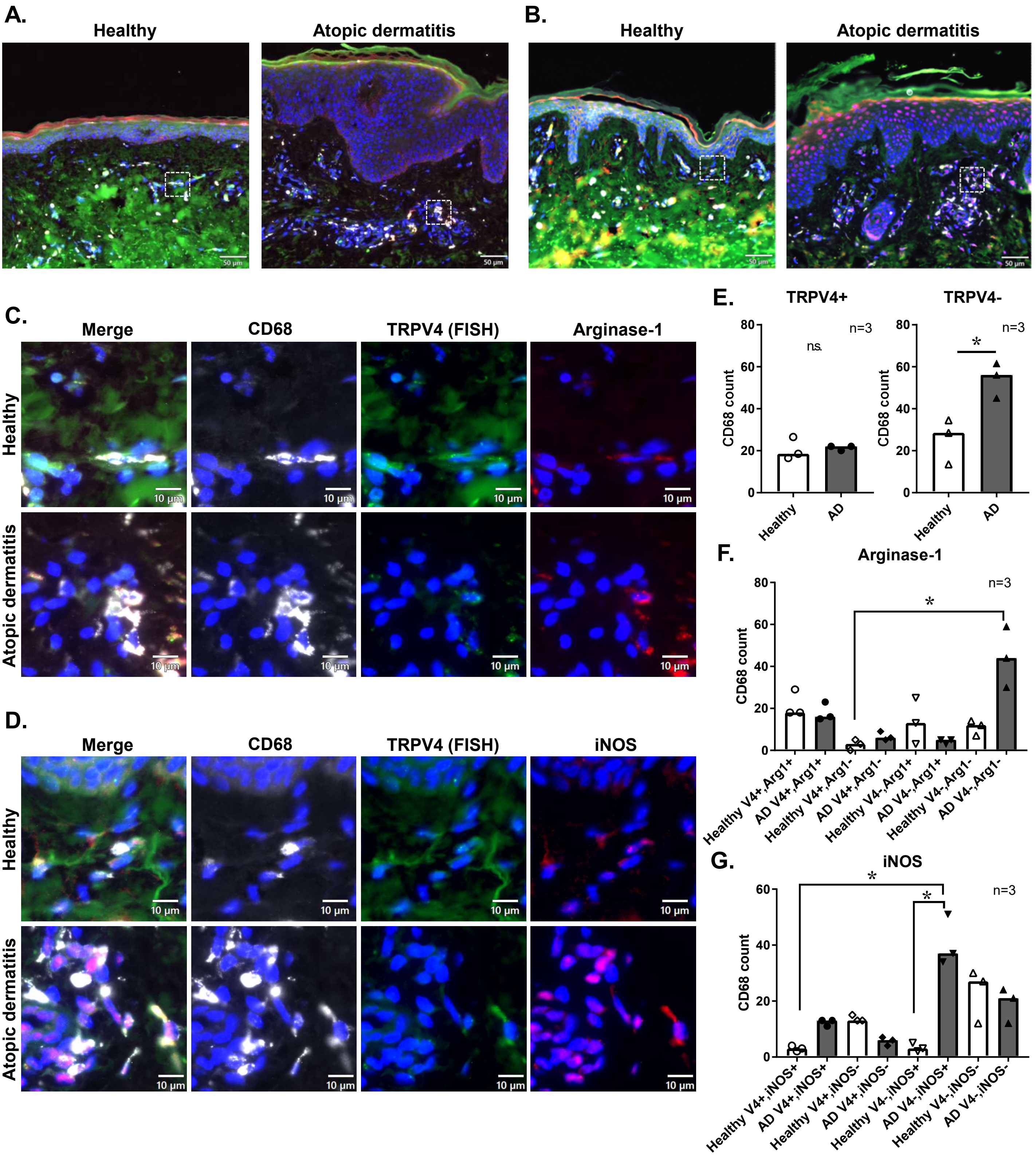

Figure S4. Downregulation of TRPV4 increases iNOS-positive macrophage presence during atopic dermatitis. (A, C) Representative fluorescent images of CD68 (white), TRPV4 (green, FISH), arginase-1 (red), and DAPI-stained nuclei in healthy and atopic dermatitis dermis (scale bar = 50 μm). **(C)** The rectangular areas (white dashline) were magnified (scale bar = 10 μm). **(B, D)** Representative fluorescent images of CD68 (white), TRPV4 (green, FISH), iNOS (red) and DAPI-stained nuclei in healthy and atopic dermatitis dermis (scale bar = 50 μm). **(D)** The rectangular areas (white dashed line) were magnified (scale bar = 10 μm). **(E)** TRPV4 expression in dermal CD68-positive macrophages in healthy and atopic dermatitis dermis. TRPV4-positive and TRPV4-negative populations in dermal macrophages were quantified. Welch's t test, *, $p<0.05$, n=3 donors. **(F, G)** The number of CD68-positive macrophages with each characteristic, **(F)** Arginase-1 and **(G)** iNOS. Three independent healthy volunteers and three independent atopic dermatitis skin specimens were examined. *, $p < 0.05$, Kruskal-Wallis test.
